# Post-transcriptional capping generates coenzyme A-linked RNA

**DOI:** 10.1080/15476286.2023.2288740

**Published:** 2023-11-30

**Authors:** Krishna Sapkota, Jordyn K. Lucas, Jarrett W. Faulkner, Matt F. Lichte, Yan-Lin Guo, Donald H. Burke, Faqing Huang

**Affiliations:** a Department of Chemistry and Biochemistry, University of Southern Mississippi, Hattiesburg, MS, USA; b Department of Biochemistry, University of Missouri, Columbia, MO, USA; c Bond Life Sciences Center, University of Missouri, Columbia, MO, USA; d Department of Cell and Molecular Biology, University of Southern Mississippi, Hattiesburg, MS, USA; e Department of Molecular Microbiology & Immunology, University of Missouri, Columbia, MO, USA

**Keywords:** CoA-RNA, PPAT, post-transcriptional CoA-capping, cofactor-RNA conjugates, CoA-RNA CaptureSeq

## Abstract

NAD can be inserted co-transcriptionally via non-canonical initiation to form NAD-RNA. However, that mechanism is unlikely for CoA-linked RNAs due to low intracellular concentration of the required initiator nucleotide, 3’-dephospho-CoA (dpCoA). We report here that phosphopantetheine adenylyltransferase (PPAT), an enzyme of CoA biosynthetic pathway, accepts RNA transcripts as its acceptor substrate and transfers 4’-phosphopantetheine to yield CoA-RNA post-transcriptionally. Synthetic natural (RNAI) and small artificial RNAs were used to identify the features of RNA that are needed for it to serve as PPAT substrate. RNAs with 4–10 unpaired nucleotides at the 5’ terminus served as PPAT substrates, but RNAs having <4 unpaired nucleotides did not undergo capping. No capping was observed when the +1A was changed to G or when 5’ triphosphate was removed by RNA pyrophosphohydrolase (RppH), suggesting the enzyme recognizes pppA-RNA as an ATP analog. PPAT binding affinities were equivalent for transcripts with +1A, +1 G, or 5’OH (+1A), indicating that productive enzymatic recognition is driven more by local positioning effects than by overall binding affinity. Capping rates were independent of the number of unpaired nucleotides in the range of 4–10 nucleotides. Capping was strongly inhibited by ATP, reducing CoA-RNA production ~70% when equimolar ATP and substrate RNA were present. Dual bacterial expression of candidate RNAs with different 5’ structures followed by CoA-RNA CaptureSeq revealed 12-fold enrichment of the better PPAT substrate, consistent with *in vivo* CoA-capping of RNA transcripts by PPAT. These results suggest post-transcriptional RNA capping as a possible mechanism for the biogenesis of CoA-RNAs in bacteria.

## Introduction

Covalent modification of cellular RNA is a critical determinant of the biological functions of those transcripts. Their fate is governed by the dynamic interplay of ‘writer’ proteins that install the modifications, ‘reader’ proteins that detect and respond to them, and ‘eraser’ proteins that remove them. The canonical 7-methylguanosine (m^7^G) cap is a ubiquitous feature of eukaryotic mRNA and is responsible for efficient mRNA translation via recruitment of translational components. Cofactor-modified RNAs with cap-like structures are a relatively new addition to this group. Although bacterial transcripts are not typically associated with 5’ caps, this view changed as Gram negative *Escherichia coli* and Gram positive *Streptomyces venezuelae* were both reported to cap some of their RNAs with metabolic cofactors NAD^+^, CoA, and CoA-thioesters at the 5’ end [1,2].

Studies on the functions, diversity, and mechanism of capping are illuminating the biological significance of these non-canonical caps [3–10]. For example, NAD^+^ capping in *E. coli* was found to protect RNA from RNase E mediated degradation [11], while in eukaryotes NAD-ylation promoted RNA decay by DXO-mediated deNAD-ylation [4]. Both co-transcriptional and post-transcriptional mechanisms have been proposed to explain RNA capping with NAD^+^ and 3’-dephospho-CoA (dpCoA) [2,12,13]. Given suitable promoters with A in the +1 position, several RNA polymerases can initiate transcription *in vitro* with adenosine cofactors such as NAD^+^, FAD, and dpCoA, including T7 RNAP, *E. coli* RNAP and human mitochondrial RNAP [12–15]. Moreover, T7 RNAP was also shown to initiate transcription efficiently with several non-biological adenosine containing molecules *in vitro* [14,16,17]. These findings substantiate the co-transcriptional mechanism of capping, essentially via competition with the canonical NTP initiator nucleotide.

Mechanistic investigation into transcription initiation by non-canonical initiator nucleotides (NCINs) has focused on NAD^+^-linked RNAs, fuelled in part by the availability of capture methods for these RNA species [11,18]. *E. coli* RNAP was shown to initiate transcription with NAD^+^ under a consensus promoter HRR**A**_+1_SWW [19]. This mode of NAD-ylation is highly efficient and yields up to 15% of NAD-linked transcripts *in vivo* under growth conditions that promote high [NAD^+^]/[ATP] ratios [12], such as stationary phase, when the [NAD^+^]/[ATP] ratio can be higher than 2:1. The Km value of NAD^+^ utilization by *E. coli* RNAP (0.38 mM) is an order of magnitude lower than the intracellular NAD^+^ concentration, which typically fluctuates between 4 and 7 mM in *E. coli* [20]. Although ATP is the preferred substrate for *E. coli* RNAP (Km = 0.09 mM), the comparable concentrations and Km values of these substrates make it feasible for *E. coli* RNAP to incorporate NAD^+^ into the +1 position of RNA transcripts. Thus, a co-transcriptional mechanism could account for much of the NAD-RNA found in cells. Further supporting this notion, Cahová et al. reported around 26% of sRNA RNAI to be capped with NAD in the absence of de-capping enzyme NUcleoside DIphosphate linked to X (NUDIX) phosphohydrolase NudC [11]. The situation for CoA-RNA is dramatically different from NAD-RNA, as the intracellular concentration of dpCoA is approximately 100–200 times lower than that of NAD^+^ [21,22], making transcription initiation with dpCoA very unlikely. Thus, to the extent that CoA-RNA transcripts exist naturally or can be engineered to form within bacterial transcriptomes, they are more likely to arise from post-transcriptional mechanisms.

**Figure 1.**
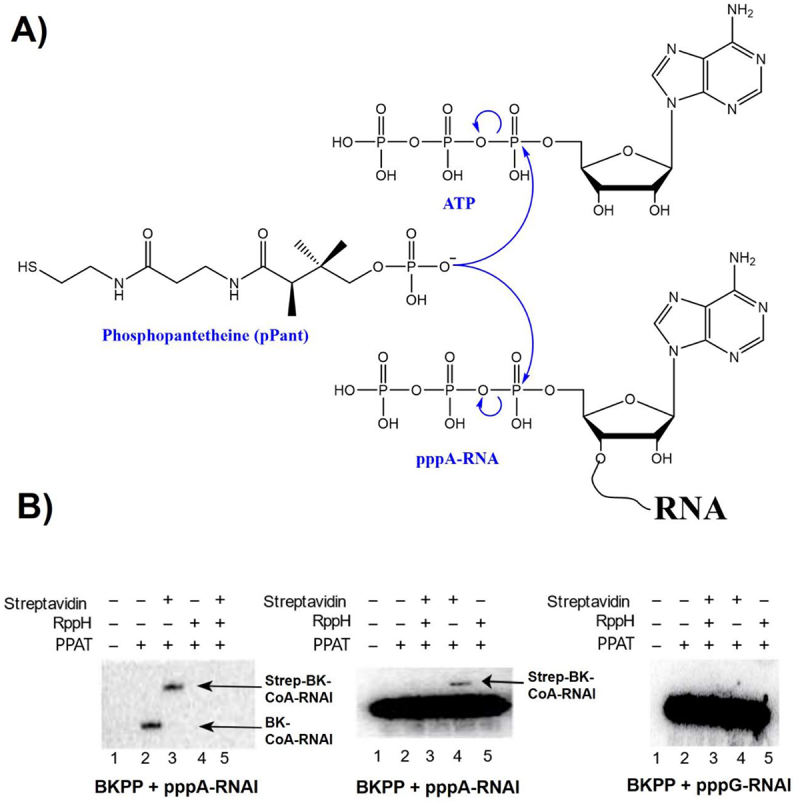
CoA-RNA synthesis by PPAT. (*A*) analogous reactions of pPant + ATP and pPant + ATP-RNA produce dpCoa (top) and CoA-RNA (below). (*B*) *in vitro* capping assays identify the necessary conditions for PPAT-catalyzed BK-CoA-RNA synthesis from reaction of ^14^C-labelled BKPP (a pPant analog, **scheme S1**) with unlabelled pppA-RNAI (left panel), ^32^P-labelled pppA-RNAI (middle panel), and ^32^P-labelled pppG-RNAI (right panel). In the left panel, only BK-capped RNAI but unreacted RNAI is visible. Streptavidin-BK-CoA-RNAI complex migrates more slowly than BK-CoA-RNAI. In the middle and right panels, both RNAI and BK-capped RNAI are visible, but BK-capped RNAI is not resolved from RNAI in the absence of streptavidin (middle panel, lane 2). Formation of streptavidin-BK-capped RNAI complex allows for BK-capped RNAI separation and detection (middle panel, lane 4). RppH treatment of pppRNA removes its 5’ pyrophosphate to yield pRNA, which does not react with PPAT.

CoA is an indispensable metabolic cofactor participating in diverse acyl transfer reactions, from the TCA cycle and fatty acid metabolism to metabolite biosynthesis and gene regulation. In *E. coli*, the *de novo* synthesis of CoA involves five enzymatic reactions starting with the phosphorylation of pantothenic acid (vitamin B5) by pantothenate kinase PanK (CoaA). The CoaBC complex converts the resulting phosphopantothenate to phosphopantetheine (pPant) by sequential cysteinylation and decarboxylation. Adenylation of pPant by phosphopantetheine adenylyltransferase PPAT (CoaD) yields dpCoA (Figure 1A, top), which is then phosphorylated at 3'-OH by CoaE, completing the pathway.

Several of the CoA biosynthetic enzymes – including PanK and PPAT – exhibit relaxed substrate specificities that allow a broad range of modifications on pantetheine, enabling enzymatic synthesis of diverse CoA analogs [23–25]. Due to the critical requirement of CoA in metabolism, these analogs have proven useful for the development of novel therapeutics, including antibiotics [26,27]. We asked whether PPAT’s ability to accept non-canonical substrates extends beyond pantetheine nucleophile analogs to include ATP-RNAs as ATP acceptor analogs, thus providing a post-transcriptional capping mechanism for the biosynthesis of CoA-RNAs.

We report here that PPAT accepts ATP-RNA in place of ATP, yielding CoA-RNA *in vitro* (Figure 1A, bottom). We have characterized the essential structural features of the substrate RNA at the 5' terminus and determined that a 5' triphosphate, a 5'-terminal adenosine, and a minimum of four unpaired nucleotides must be present at the RNA 5' terminus for the PPAT-mediated pPant capping. Furthermore, using the *in vitro* substrate requirements as a guide, we expressed in *E. coli* RNA D2* as a poor PPAT RNA substrate and RNA D7* as a good PPAT RNA substrate. These RNAs were then partitioned, sequenced, and quantified. Interestingly, RNA samples that underwent a thiol-partition method to isolate CoA-RNAs showed more than a 10-fold increase in D7*:D2* RNA ratios as compared to the total RNA samples, consistent with a model in which D7* RNA is preferentially capped by pPant via PPAT catalysis *in vivo* to generate CoA-RNAs. Overall, both the *in vitro* and *in vivo* experiments suggest that bacterial CoA-RNAs may be generated as a product of post-transcriptional capping by PPAT, making PPAT a potential ‘writer’ of this unusual post-transcriptional modification of RNA.

## Results

### Coli PPAT enzyme can cap native RNA in vitro to form CoA-RNA

To test the hypothesis that CoA-RNA can be generated by posttranscriptional capping (Figure 1A, bottom) via a ‘writer’ enzyme, we considered several factors, including the most probable enzyme, a natural small and abundant RNA, and a suitable method for direct CoA-RNA detection. Since the hypothesized capping reaction is analogous to dpCoA biosynthesis (Figure 1A, top), PPAT is the most probable ‘writer’ enzyme for our consideration. Recombinant *E. coli* PPAT was overexpressed and purified (***SI Appendix*** **Fig. S1**), and its activity for synthesis of genuine dpCoA from pPant and ATP was monitored by HPLC (***SI Appendix*** **Fig. S2**). Based on the reaction rate (slope of the plot of product vs time), k_cat_ was calculated to be 30.8 min^−1^ (see kinetics description in ***SI Appendix***), which is about one-third of the reported values of 83.4 and 95.4 min^−1^ for pPant and ATP, respectively. The difference was more likely due to the different conditions for the kinetic experiments [28], instead of differing PPAT activity of different preparations.

RNAI is an antisense RNA (108 nt, beginning with ACAGU …) that regulates the replication and copy number of some plasmids in *E. coli*, such as ColE1 [29,30], and it is the most abundant NAD-ylated RNA in *E. coli* [11]. We therefore chose RNAI as the target RNA to test whether it can serve as a PPAT substrate and be capped with pPant to yield CoA-RNA *in vitro*. To facilitate transcription by T7 RNA polymerase [14], a G was inserted after initiating A to generate 109 nt RNA (***SI Appendix*** **Fig. S3**).

A direct and unambiguous method for CoA-RNA detection from the reaction (Figure 1A, bottom) is essential to test our hypothesis, particularly so when considering the likely low reaction yield by PPAT. Based on our prior experience with CoA-RNA transcripts formed either co-transcriptionally or through the action of self-capping ribozymes [14,31,32], the transfer of pPant to RNAI is expected to slow its migration on PAGE so that it moves as N + 1 RNA. However, the large size of RNAI makes it challenging to resolve 110 nt (CoA-G-RNAI) from unreacted 109 nt RNA (AG-RNAI) unambiguously by PAGE. Internal radiolabeling of RNA by using ^32^P is also not ideal, since the gel mobility of CoA RNA-product is equivalent to 3'-extended N + 1 RNA, which may alternatively be generated by the transcription itself [33]. Furthermore, the expected low band intensity of product CoA RNA would be buried within the strong RNA band, due to their close proximity on the gel. To make the visualization of the CoA-RNA product simple and straightforward, we designed and synthesized pPant analog BKPP tagged with both ^14^C and biotin (***SI Appendix*** **Scheme S1**) so that only the RNA that has the BKPP cap will be visible upon exposure to a phosphor storage screen.

When ^14^C-containing BKPP and unlabelled synthetic RNAI with +1A were used as PPAT substrates, we observed a transfer of BKPP to RNA, yielding CoA-RNAI analog *in vitro* (Figure 1B, left panel, lane 2), compared with the negative control (Figure 1B, left panel, lane 1). The addition of streptavidin retarded the electrophoretic mobility of the product, further confirming that PPAT successfully capped RNAI with BKPP (Figure 1B, left panel, lane 3). We did not observe capping of RppH-treated RNAI (Figure 1B, left panel, lanes 4 & 5). RppH treatment removes 5' pyrophosphate from pppRNAI to yield pRNAI, which did not meet the requirements to be an enzyme substrate. Similar experiments were performed using^14^C-labelled BKPP and ^32^*P*-labelled RNAI. The expected reaction product was not resolved from RNAI and became invisible under the strong ^32^P radioactivity (Figure 1B middle panel, lane 2). However, addition of streptavidin led to a new band (Figure 1B middle panel, lane 4), indicating RNAI capping by BKPP. To further investigate whether an ATP at the +1 position is a strict requirement, we prepared RNAI with GTP at the +1 position by *in vitro* transcription under T7 φ6.5 promoter. When pppG-RNAI was used as a PPAT substrate, the enzyme did not catalyse the transfer of BKPP to RNA (Figure 1B, right panel, lane 4). These experiments established that RNA requires a 5' triphosphate and an adenosine at the +1 position to serve as a PPAT substrate and to be capped with BKPP to yield a CoA-RNA analog BK-CoA-RNA.

### PPAT accepts 5 mer RNA as its substrate and caps with pPant to form genuine CoA-RNA

**Figure 2.**
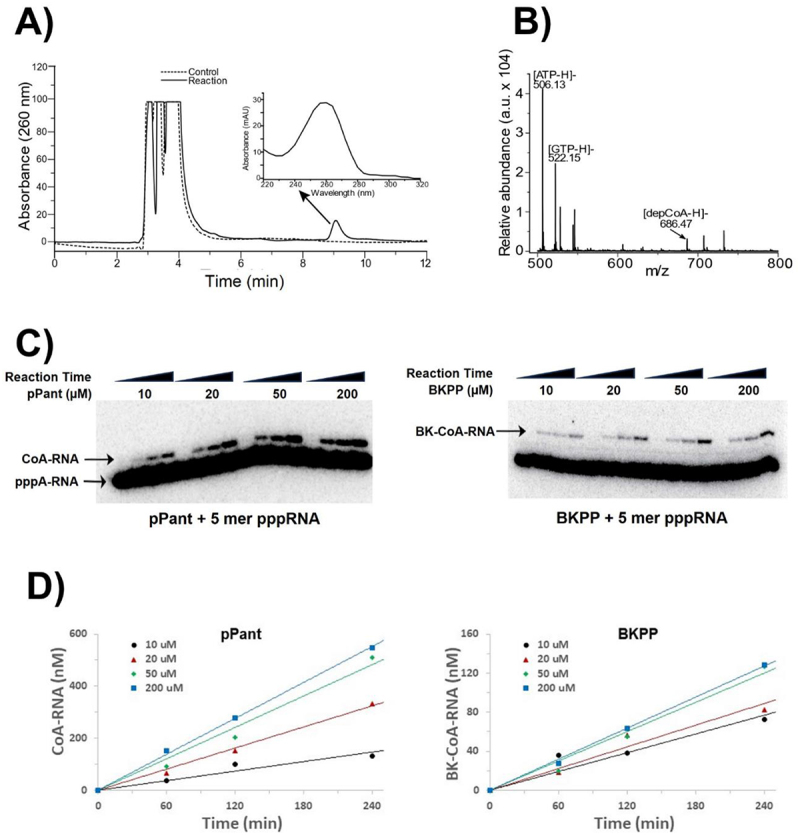
Characterization of CoA-RNA synthesis by PPAT. (*A*) HPLC and (*B*) mass spectrometry were used to analyze nuclease P1 digestion products of reactions performed with (solid line) or without (dashed line) PPAT for 4 h. Panel *B* shows a product peak having the same retention time as genuine dpCoa and a characteristic ‘adenosine’ UV spectrum (inset, obtained from an online PDA detector). The observed m/z ratio matches the expected value for dpCoa (*B*). (*C*) *in vitro* capping kinetic assays were carried out using 10 µM internally ^32^P-labelled 5 mer pppRNA (***SI Appendix*** **fig. S5**), 500 nM PPAT, and either pPant (left panel) or BKPP (right panel). pPant and BKPP substrates were used at concentrations of 10, 20, 50 and 200 µM, and reactions were carried out for 0, 60, 120, or 240 min. (*D*) plots of capped RNA product concentrations vs reaction time for CoA-RNA (left) and BK-CoA-RNA (right) formation. Linear regression was used to derive the initial velocity (V_0_) at a defined substrate concentration [pPant] or [BKPP]. Since the product formation was < 5% of the total RNA, product inhibition was negligible. The V_0_ values were then used to estimate the maximum velocity at 10 µM RNA and saturating [pPant] or [BKPP], V_max_, and k_cat_ (***SI Appendix*** **fig. S6** and **kinetic** description).

To investigate post-transcriptional RNA capping by PPAT to yield genuine CoA-RNA, a 5 mer RNA of the sequence pppAGGAA was prepared by abortive transcription *in vitro* and purified by ion pairing reverse phase HPLC (***SI Appendix*** **Fig. S4**). Authentic pPant was synthesized using recombinant PanK purified in house (***SI Appendix*** **Fig. S1**). After incubating pppAGGAA and pPant with or without PPAT, both reactions were digested to mononucleotides with nuclease P1 and separated by ion pairing reverse phase HPLC. The P1-digested PPAT reaction produced an extra peak on HPLC having the same retention time as that of authentic dpCoA and an adenosine-like UV signature (Figure 2A). When the nuclease P1 treated samples (the control and the reaction) were analysed by MALDI-ToF, we observed a peak in the PPAT reaction having m/z of 686.47, corresponding to [dpCoA-H]^−^ (expected m/z = 686.14) (Figure 2B). These experiments concluded that PPAT catalysed the transfer of pPant onto the 5' end of 5 mer RNA to yield CoA-RNA *in vitro*. Therefore, pppAGGAA is an active ATP analog, and PPAT is able to catalyse the coupling reaction between pPant and pppAGGAA to produce CoA-GGAA. The reaction is analogous to *in vivo* dpCoA biosynthesis (Figure 1A).

### PPAT transfers BKPP and pPant to 5’ terminus of RNA with similar rates

Having established that PPAT can accept pppA-RNA as a substrate in place of ATP and transfer both BKPP and pPant to RNA 5' terminus, we next compared the kinetics of capping with the synthetic BKPP and the natural substrate pPant. The same small RNA as above was prepared with internal radiolabel by abortive *in vitro* transcription in the presence of [α-^32^P] ATP (***SI Appendix*** **Fig. S5**) and gel purification. For small substrate RNAs, the substrate (pppRNA) and the product (CoA-RNA) can be easily resolved by denaturing PAGE. Therefore, after incubating substrates with PPAT for the specified times at 37°C, reactions were quenched by freezing at −20°C, and products were separated by 20% denaturing PAGE (Figure 2C). Product concentrations were calculated from band intensities and plotted against reaction time (Figure 2D). The reaction rates were obtained by linear regression, and resulting values were used in calculating Michaelis–Menten parameters (***SI Appendix*** **Fig. S6**) to obtain the maximum velocity of 2.4 and 0.6 nM/min for pPant and BKPP, respectively, at 500 nM PPAT, 10 µM RNA and saturating pPant/BKPP (see kinetics description in ***SI Appendix***). Therefore, the synthetic analog BKPP is about 4 times slower than the natural pPant. Since K_m_^ATP^ for PPAT is 220 µM [28], we surmise that K_m_^ATP-RNA^ is similar to or higher than 220 µM, making it impractical for its direct measurement. However, assuming equivalent values for K_m_^ATP^ and K_m_^ATP-RNA^, we can calculate V_max_ for PPAT-catalysed dpCoA synthesis under our reaction conditions to be 55 nM/min, and k_cat_ = 0.11 min^−1^ (see kinetics description in ***SI Appendix***). Therefore, PPAT-catalysed dpCoA synthesis is likely 280 times more efficient than CoA-RNA synthesis under the same conditions, indicating a strong preference for the natural substrate ATP over its ATP-RNA analog.

### The ability of RNA to serve as PPAT substrate is determined by the structure at its 5’ terminus

Even though the PPAT-mediated chemistry is identical with pppA (ATP) and pppA-RNA (Figure 1A, ATP-RNA, e.g. RNAI and 5 mer RNA), we reasoned that access to the enzyme’s active site may be blocked if a large stem-loop structure is present very close to the 5' terminus. The 5 mer RNA used above to study the kinetics of pPant and BKPP capping does not fold into any significant secondary structure. In contrast, the 109 mer *E. coli* RNAI has two structures as predicted by Mfold (***SI Appendix*** **Fig. S3**). Both structures have 3 stem-loops and a stretch of either 4 or 10 unpaired nucleotides at the 5' terminus (including the appended dinucleotide).

**Figure 3.**
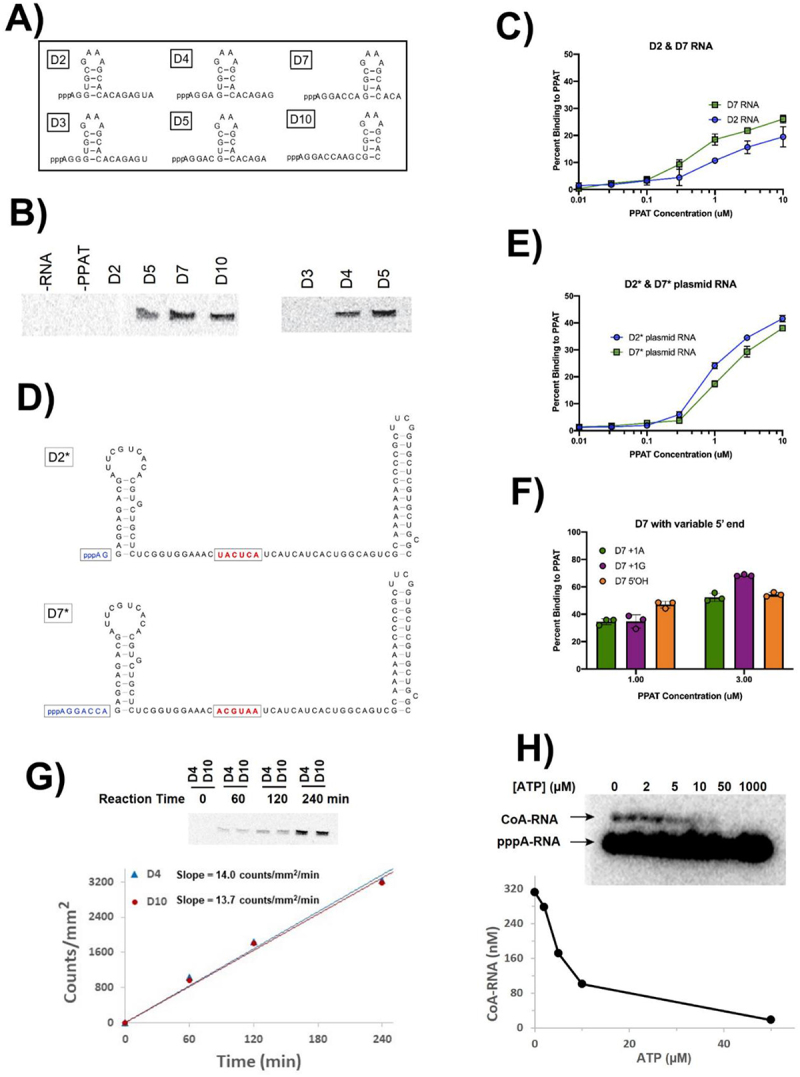
Requirements for CoA-RNA formation by PPAT. (*A*) six model RNA sequences (22 nucleotides each) containing a stable stem-loop structure with variable distances from the 5’ termini. (*B*) BK-CoA-RNA formation using ^14^C-labelled BKPP and unlabelled pppRNA from (*A*). The enzyme accepts D4, D5, D7, and D10 RNA as substrates but not D2 or D3 RNA. (*C*) relative PPAT binding affinities for D2 and D7 RNA were determined in nitrocellulose filter binding assays using trace amounts of radiolabelled RNA and the indicated PPAT concentrations; *n* = 3 independent measurements for all binding assays. (*D*) sequences and predicted secondary structures (mfold) for D2* and D7* RNAs. Red nucleotides are indices used for *in vivo* activity evaluations (fig. 4). (*E*) evaluation of D2* and D7* RNA binding to PPAT as in (*C*); *n* = 3. (*F*) evaluation of D7, D7 + 1 G, and D7 5’ OH RNA binding to 1 or 3 μM PPAT. (*G*) D4 and D10 relative rate comparison by incubating 10 μM D4 and D10 RNA at 37°C with 200 μM BKPP and 500 nM PPAT for 1–4 hr. Products were separated by PAGE and visualized by phosphorimaging. The band intensity (counts/mm^2^) was plotted against incubation time, and linear regression was used to derive the slope (counts/mm^2^/min), yielding similar reaction rates. (*H*) ATP inhibition of CoA-RNA synthesis using 500 nM PPAT, 200 μM pPant and 10 µM ^32^P-labelled 5 mer pppRNA, 4 h at 37°C.

To study the effect of RNA structure near the 5' terminus on CoA capping, we designed six different 22 mer RNAs that are predicted to have only one thermodynamically favourable secondary structure, with a stable stem-loop at systematically varied distances from the 5' terminus. Specifically, D2 RNA has a stretch of two unpaired nucleotides before the stem-loop, while D3, D4, D5, D7 and D10 RNAs have stretches of 3, 4, 5, 7 and 10 unpaired nucleotides at their 5' ends, respectively (Figure 3A). To simplify product visualization and analysis, BKPP was used as pPant analog so that only the RNAs that serve as PPAT acceptor substrates are labelled with [^14^C]. Gel images of reaction products showed that D4, D5, D7 and D10 RNAs, which have ≥4 unpaired nucleotides at the 5' terminus, underwent PPAT-catalysed BKPP capping. However, the D2 and D3 RNAs, which have shorter unpaired stretches of ‘AG’ (2 nt) or ‘AGG’ (3 nt) at their 5' termini, did not undergo capping (Figure 3B). These results established that an RNA needs a stretch of at least 4 unpaired nucleotides at the 5' terminus to undergo PPAT mediated capping to form CoA-RNA.

### RNA binding affinity to PPAT is not determined by the 5’ terminus

The BKPP incorporation results above establish that CoA capping by PPAT requires RNAs to have an unstructured 5' terminus and a 5' triphosphate. We wondered whether RNA binding to PPAT occurred primarily via interactions in the active site and whether RNAs lacking the proper chemical and structural features were capable of binding to PPAT. Therefore, we radiolabeled RNA substrates and tested their binding to PPAT using a nitrocellulose filter binding assay. The D7 RNA bound to PPAT only marginally better than D2 RNA, especially at the µM PPAT concentration used in the capping reactions, even though D2 does not meet the required 5' terminus structural requirements for the capping reaction (Figure 3C). Longer RNA substrates D2* and D7* (102–107 nt) were designed to mimic the key features of D2 and D7 RNAs: +1A, same single-stranded 5' termini with 2 or 7 unpaired 5' nucleotides, followed by stable stem (Figure 3D). No significant difference in binding to PPAT was observed between these two RNAs (Figure 3E). Finally, the potential importance of the chemical composition of the 5' terminus was evaluated by measuring binding at 1.0 and 3.0 µM PPAT for RNAs that carried either pppG or HO-A in the +1 position. Neither of these modifications reduced RNA binding to PPAT (Figure 3F), even though both are incompatible with capping by PPAT. We conclude that overall RNA binding to PPAT does not occur solely in the active site, and that productive interactions that lead to capping are likely controlled by local positioning effects in the active site rather than by overall affinity.

### The number of unpaired nucleotides at the 5’ terminus does not affect capping kinetics

We next examined how capping kinetics are affected by the number of unpaired nucleotides at the 5' terminus of substrate RNA. D4 RNA, which meets the minimum requirements to undergo capping, and the D10 RNA, which has the maximum number of unpaired nucleotides at the 5' terminus among our designed RNAs, were used as PPAT acceptor substrates. Band intensities (product yields) increased with time for both D4 and D10 RNAs, as expected (Figure 3G). Linear regression gave essentially the same reaction rates of 14.0 and 13.7 counts/mm^2^/min for D4 and D10, respectively. These data clearly indicate that the rate of PPAT-catalyzed RNA capping by BKPP is independent of the number of free nucleotides at 5' unpaired region for the range of 4–10 nt. If the minimum requirement of ≥4 nucleotides at 5' unpaired region is met, the reaction proceeds with the same speed regardless of the number of unpaired nucleotides present.

### ATP inhibits the transfer of pPant to pppA-RNA by PPAT

Since PPAT appears to prefer ATP as acceptor substrate over RNA, we investigated how the presence of ATP affects pPant transfer to pppA-RNA. The same 5 mer pppA-RNA as above was incubated with PPAT and 200 µM pPant in the presence of varying concentrations (0–1000 µM) of ATP (Figure 3H, top), and reaction yields were plotted against ATP concentration (Figure 3H, bottom). Product yield decreased in the presence of ATP, with half-maximal inhibition around 5 µM ATP. No CoA-RNA product was observed when ATP concentration was increased to 1 mM. These results imply that posttranscriptional CoA-RNA synthesis would experience significant competition from intracellular ATP, given that the intracellular concentration of ATP in *E. coli* is 1–5 mM [34].

### Differential in vivo capping of D2* and D7* RNA substrates by PPAT

To determine whether PPAT was capable of *in vivo* capping, RNA substrates D2* and D7* were cloned into a plasmid for constitutive dual expression under separate strong promoters along with the *coaD* gene for inducible expression of PPAT. The two RNAs are identical except for their 5' termini and an internal 6 nt index to enable differentiation of the two transcripts by RNASeq (Figure 3D& ***SI Appendix*** **Table S1**). We reasoned that if PPAT acts upon these RNAs within bacterial cells, then it will cap D7* transcripts more efficiently than D2* transcripts, with the consequence that the ratio of (CoA-D7*):(CoA-D2*) will be enriched for D7* and depleted for D2* relative to the (D7*):(D2*) ratio for total RNA.

**Figure 4.**
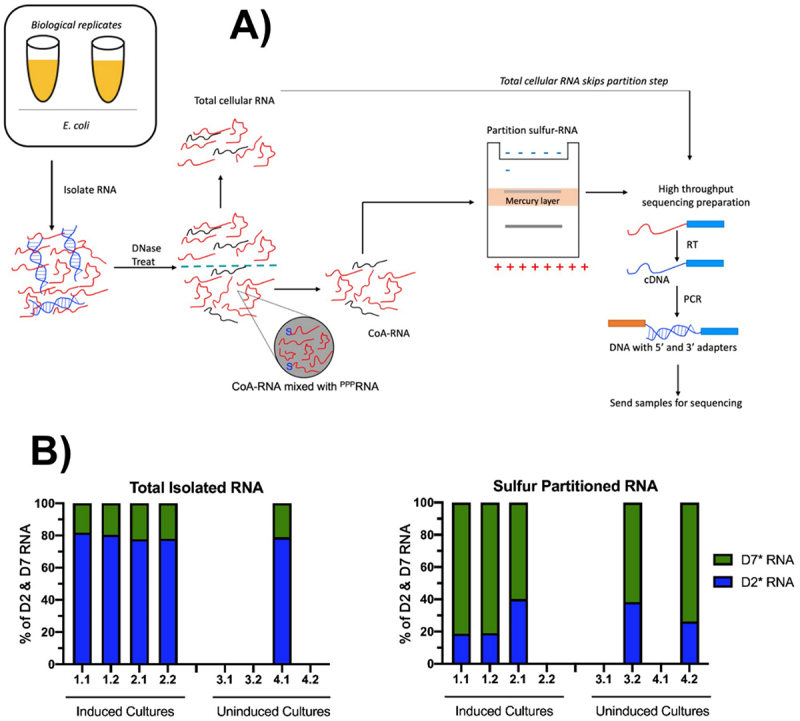
**Capturing cellular CoA-RNAs capped *in vivo***. (*A*) schematic of CoA CaptureSeq method summarizing the various steps to isolate, prepare, partition, and sequence CoA-RNAs from total RNA. RNA isolated from duplicate bacterial cultures were each separated into two technical replicates prior to DNase treatment. Sulphur-containing samples were partitioned on an APM gel, then RT-PCR amplified for high-throughput sequencing. (*B*) reads corresponding to D2* (blue) and D7* (green) RNAs were identified by their respective index sequences (**fig. 3*D***), and their relative proportions in each sample were determined for total RNA (left) and CoA-RNA partitioned (right) samples. Samples with fewer than 2,000 total unique processed reads (***SI Appendix*** **Tables S1 & S2**) were excluded from these data sets.

A ‘CoA CaptureSeq’ method was developed to determine these ratios (Figure 4A). Briefly, total RNA was isolated from four cultures of transformed *E. coli* carrying a dual expression plasmid (two biological replicates of induced cultures and two of uninduced cultures). Each of the four samples was split for processing as technical replicates. Contaminating DNA was removed with DNase and each sample was split again for processing two different ways to recover either total RNA (no partition step) or sulphur-containing RNA (partition on a tri-layer mercury gel containing acryloylamino-phenylmercuric chloride, APM, as previously described) [35–38]. Following elution from the APM layer of the gel and ethanol precipitation, both total RNA and sulphur-containing RNAs were reverse transcribed, PCR amplified and prepared for high-throughput sequencing (***SI Appendix*** **Tables S1 & S2**). Read counts for D7* and D2* RNAs were determined from their respective indices, and these values were converted to ratios. Of the 16 sampled populations, six had insufficient reads and were discarded. Ten populations each yielded >2000 processed reads that could be unambiguously identified as D7* or as D2* (five populations for total RNA and five for sulphur-containing RNA) and were used in the analysis (***SI Appendix*** **Tables S2 & S3**).

Clear trends emerged from this dataset. In the total RNA samples, D7* consistently made up around 20% of the read counts whereas D2* comprised around 80% (Figure 4B, left), corresponding to a 1:4 ratio of D7*:D2*. In contrast, for the sulphur-containing RNA samples recovered from APM gels, which should contain the CoA-capped RNAs, D7* made up ~71% of the read counts (Figure 4A, right), corresponding to a 2.45:1 ratio of CoA-D7*:CoA-D2*. Brief induction of plasmid PPAT expression did not noticeably impact the amount of D7* RNA that was capped, although it remains to be seen whether longer induction might generate a greater difference by producing more CoaD enzyme. These data show more than a 12-fold increase in D7*:D2* RNAs between the thiol RNA samples and total RNA samples, exactly in the direction predicted based on the relative suitability of these two RNAs for capping by intracellular PPAT.

## Discussion

By using *in vitro* transcribed RNAs of diverse sizes (5 to 109 nt), we have demonstrated that RNA can be capped post-transcriptionally to produce CoA-RNA by the activity of PPAT. The substrate RNA requires three distinct features at the 5' terminus: a triphosphate group on the terminal nucleotide, adenosine in the +1 position (Figure 1B), and a stretch of four or more unpaired nucleotides (Figure 3A). These findings represent a possible novel mode of post-transcriptional RNA capping as demonstrated by our CoA-CaptureSeq data (Figure 4). Although PPAT was known to accept a broad range of modifications to the pantetheine/pantothenate substrate [24,25,39], our results demonstrated its ability to take modifications on the ATP substrate – albeit at substantially reduced efficiency – since pppA-RNA can be considered as an ATP analog that carries a bulky 3' modification in the form of a long stretch of nucleotides. This atypical activity of PPAT unambiguously generated CoA-RNA *in vitro*. In principle, similar PPAT-catalysed reactions could occur within bacteria under certain circumstances for RNAs that meet the three requirements mentioned above and contribute to CoA-RNA biogenesis *in vivo*.

The activity of PPAT is regulated by free CoA through feedback inhibition. To achieve this regulation, the enzyme binds both CoA and dpCoA using distinct binding modes but at somewhat overlapping sites (***SI Appendix*** **Fig. S7**). While dpCoA occupies the same space and orientation as those of pPant and ATP, the pantetheine arm and the adenosine group of CoA are oriented differently from those of dpCoA [40–42]. This difference probably prevents the enzyme’s activity on CoA. Indeed, while PPAT can act upon dpCoA to catalyse the reverse (pyrophosphorolysis) reaction (dpCoA + PP_i_ → pPant + pppA), it is unable to catalyse pyrophosphorolysis of CoA (CoA + PP_i_ → pPant + pppAp) [43], again suggesting that local orientation within the active site governs reactivity. In addition, CoA was reported to bind differently to two trimers of PPAT hexamer to achieve allosteric feedback inhibition, with one CoA occupying a different site and orientation from those of dpCoA (***SI Appendix*** **Fig. S7 *D***) and the other CoA having the electron density of only the pPant portion in opposite direction (***SI Appendix*** **Fig. S7 *A*, S7*B***), indicating disordered AMP portion [42]. Since CoA is not a substrate for PPAT [43], neither of its two binding modes is compatible with the PPAT catalytic mechanism. When the binding modes above are used to model pppA-RNA binding to PPAT, the additional 3' phosphate and nucleotides cannot fit into either site of ATP, dpCoA, or CoA (***SI Appendix*** **Fig. S7**). It is intriguing, then, to wonder how PPAT is able to bind pppA-RNA at the active site in the correct spatial orientation to effect catalysis. We suggest that the interaction of PPAT with the terminal pppAp of pppA-RNA may induce a conformational change to accommodate pppA-RNA at the active site with a correct orientation to produce the weaker activity on pppA-RNA relative to ATP. Although CoA is not a substrate for PPAT, we surmise that pppAp (unknown molecule) and pppApp [44] may be substrates to yield genuine CoA and CoAp (unknown molecule), respectively, by reacting with pPant at a slower rate than ATP-pPant reaction. This prediction may be extended further to other pppAy (y = chemical group, such as alkyl, aromatic, amino, thiol, sugar, amino acid, peptide, lipid, nucleotide) to produce different CoA analogs CoAy. Therefore, PPAT is an enzyme with a high degree of plasticity, capable of accepting both pPant analogs (modification at the thiol end) and ATP analogs (modification at the 3' end) to produce three series of CoA analogs, xCoA, CoAy, and xCoAy. It is tempting to speculate that a stretch of four or more unpaired nucleotides may provide the necessary flexibility to correctly position pppA-RNA and that once the +1A of RNA gets into the enzyme’s active site, the chemistry is the same as that of ATP. Interestingly, the binding data indicated no obvious difference in RNA binding related to the structure or chemical composition of the 5' terminus (Figure 3E,F). Therefore, PPAT capping requirements may be a reflection of substrate orientation and chemical reactivity in the active site, but not necessarily due to overall RNA substrate binding affinity. Ultimately, detailed structural and mechanistic studies are needed to fully uncover the molecular features of enzyme–RNA interaction.

PPAT-mediated formation of CoA-RNA in cells could be limited from competition with intracellular ATP, based on three observations. First, ATP strongly inhibited the activity of PPAT on RNA to form CoA-RNA *in vitro* (Figure 3H). Second, PPAT acts upon ATP as the acceptor substrate 280-fold faster than it does with pppA-RNA as the acceptor substrate (assuming similar Km for ATP and ATP-RNA). Third, the ATP reaction quickly forms dpCoA, which not only acts as a feedback inhibitor but also reduces the concentration of available pPant. Collectively, this inhibition may imply that PPAT mediated pPant transfer to RNA might not lead to efficient accumulation of CoA-RNA *in vivo*.

However, our CoA-RNA CaptureSeq data from *in vivo* experiments revealed a 12-fold change in D7*/D2* ratios in the thiol-containing RNA samples relative to the ratio in total RNA samples, in line with the expectation that D7* RNA should be preferentially capped with CoA by PPAT in cells. Because D2* and D7* RNAs were expressed from strong constitutive promoters, they may accumulate at high levels that aided competition with intracellular ATP and allowed for detectable capping. Furthermore, while the *in vivo* existence of CoA-RNA was first reported in 2009 [2], CoA-RNA in living cells have been reported recently [45,46]. In addition, NudC, LudL and a number of other Nudix hydrolases, such as Nudt 2,7,8,12,15,16 and 19, possess hydrolytic activity to decap CoA-RNA *in vitro* [47,48] and *in vivo* [10]. While some of the mechanistic details remain to be resolved, our *in vitro* and *in vivo* data indicate a possible role for PPAT in post-transcriptional CoA capping in bacterial cells.

Although the biological significance of CoA-RNA is not yet clear, at least four speculative models suggest themselves. First, the CoA-RNA might function as a sensor for a cell’s energy state, since growth conditions that support abundant ATP are expected to inhibit PPAT activity on RNA, preventing CoA-RNA formation, while low-ATP conditions could favour CoA-RNA. The second possibility arises from reports of thioester forms such as acetyl CoA-RNA, succinyl CoA-RNA, and malonyl CoA-RNA [2]. These CoA-thioesters are high-energy intermediates in acyl transfer reactions and their presence at the RNA 5' end could direct the RNA’s reactivity. Unlike CoA-RNA which can be generated either co-transcriptionally by RNAP or post-transcriptionally by PPAT, thioester-CoA-RNA could only be synthesized post-transcriptionally by thioesterification of CoA-RNA, since thioester-dpCoA (the corresponding transcription initiators) are not known to be present in *E. coli*. It is intriguing to speculate that CoA-RNA might be a substrate for one or more acyl CoA synthetases to drive post-transcriptional acylation of CoA-RNA into thioester-CoA-RNA. Third, CoA-RNA could be one of multiple 5' adducts formed by side reactions of enzymes originally selected for other purposes. While PPAT normally acts upon ATP, we show here that it can also accept ATPy, in which y is a bulky group in the form of ATP-RNA. Among the many cellular enzymes that use NTPs as substrates, some may also accept pppRNA, potentially forming myriad other RNA 5' adducts. Even if they form as minor side products, such modified transcripts could play heretofore unrecognized roles in cellular metabolisms. Finally, CoA-RNA might represent a molecular fossil from an RNA world in which RNA served in both genetic and catalytic capacities before the emergence of the contemporary ribonucleoprotein (RNP) world [49]. Chemically reactive moieties such as the thiol in CoA, nicotinamide in NAD^+^, and isoalloxazine in FAD – can expand the catalytic repertoire of RNA. For example, we recently described a flavin-binding RNA aptamer that shifts the reduction potential of the bound cofactor, dramatically enhancing its intrinsic reactivity relative to that of free flavin [50]. Bound organic cofactors may have similarly aided ribozyme catalytic diversity during an RNA world, especially when attached covalently to achieve the favourable entropy effect. Indeed, CoA-RNA transcripts have been used during *in vitro* selections to isolate ribozymes that promote thioester formation and aminoacylation [32,51]. Other ribozymes have been isolated that promote self-capping with pPant, FMN, and NMN to form CoA-RNA, FAD-RNA, and NAD-RNA conjugates [31]. Similar coupling reactions might have occurred during an RNA world to provide RNA an extra layer of reactivity, analogous to the cofactors of protein enzymes. With respect to the biology of CoA-RNA conjugates, it remains to be seen what mechanisms drive their production and what roles they may play in extant, engineered, or emergent biologies.

## Materials and Methods

Additional experimental details are in ***SI Appendix***.

**Expression and purification of enzymes**. Recombinant versions of CoaA and PPAT were expressed and purified from separate plasmids encoding *coaA* and *coaD* (Addgene # 50386 & 50388) [39,52] according to a modified version of our published protocol [25], then stored in 50% glycerol at −20°C until use.

**RNA transcripts**. All RNAs were generated by *in vitro* transcription from synthetic DNA templates (***SI Appendix*** **Table S1**) from Integrated DNA Technologies (IDT), using Y639F T7 RNA polymerase [53] and T7 φ2.5 promoter for +1 A or T7 φ6.5 promoter for +1 G, followed by gel or ion-pairing HPLC purification. Secondary structures were predicted by Mfold [54].

**Synthesis of pPant**. Recombinant PanK/CoaA was used to synthesize pPant. We first synthesized ox-pPant by phosphorylating pantethine in a PanK/CoaA catalysed reaction and purifying the disulphide-linked product by reverse phase HPLC. Reduction of ox-pPant by tris-carboxyethyl phosphine (TCEP) yielded pPant in high purity.

***In vitro*** **PPAT assay**. All PPAT reactions were performed at 37°C in a reaction buffer (20 mM Tris, pH 7.5, 100 mM NaCl, 10 mM KCl, 5 mM MgCl_2_) and were quenched by freezing at −20°C. Except where noted, substrate concentrations and reaction times were typically as follows: 5–10 µM RNA, 0–200 µM pPant/BKPP, 0–1 µM PPAT, for 2–4 h. Control reactions were quenched immediately (~0 min) by heating at 80°C and stored at −20°C until further processing. Reactions were loaded directly onto urea-PAGE (12%–20%), run for 1–4 hr at 15 W, dried, and exposed to phosphor screen for 1–7 days. Bands were visualized by phosphorimager and quantified by using volume analysis tool of Quantity One software (Bio-Rad).

**3' end radiolabeling RNA transcripts**. To radiolabel RNA 3’ ends for nitrocellulose filter binding assays, 100 pmol of RNA was mixed with 100 pmol of a DNA oligo with 9 complementary nucleotides flanked by a 5’GG overhang (as template for radiolabel incorporation) and a six-nucleotide unpaired 3’ overhang (to prevent extension of the template oligo) (***SI Appendix*** **Table S1**), 1 μL of 250 μCi of α^32^P dCTP (Perkin Elmer), 1X isothermal buffer (New England Biolabs), 6 mM MgSO_4_, 1.6 mM dNTP mix, and 16 U BST 3.0 DNA polymerase (New England Biolabs, ‘BST’) in a 25 μL reaction volume. BST reactions were incubated at 65°C for 1 h and heat inactivated at 80°C for 20 min. Radiolabeled transcripts were subsequently purified by ethanol precipitation and stored at −20°C until further use.

**RNA isolation from bacteria**. Using OD_600_ values, approximately equivalent numbers of bacterial cells were harvested by centrifugation at 4,000 g at 4°C for 15 min. Pellet was resuspended in 5 mL lysozyme buffer (50 mM Tris HCl pH 7.6, 250 mM NaCl, 0.1 mM EDT). Lysozyme (ThermoFisher Scientific) was added to a final concentration of 0.2 mg/mL and allowed to incubate for 10 min at room temperature. Three volumes of TRIzol (ThermoFisher Scientific) were added to the total cell lysate and vortexed before incubating on ice for 5 min. Cold (4°C) chloroform (ThermoFisher Scientific, 1/5 of the total TRIzol volume) was added and the sample was briefly vortexed to mix. Samples were centrifuged at 12,000 g at 4°C for 15 min. The top aqueous layer (~10 mL) was carefully removed to a fresh 50 mL falcon tube and 2 volumes of cold chloroform were added to remove any leftover phenol. Samples were vortexed then centrifuged at 12,000 g at 4°C for 15 min. The top aqueous layer was collected and an equal volume of cold isopropanol (ThermoFisher Scientific) was added and the sample was vortexed. Samples were incubated on ice for 5 min before being centrifuged at 16,000 g at 4°C for 30 min. The supernatant was discarded and the pellet was washed with cold 70% ethanol and centrifuged at 16,000 g at 4°C for 30 min. Pellets containing the RNA were dried and resuspended in 1 mL of MilliQ water and stored at −20°C until further use.

RNA samples were loaded onto the APM gel and run in 1X TBE at 30 W. CoA-RNAs were visualized by UV-shadow at the top APM interface and excised from the gel. Gel pieces were ‘minced’ into very small pieces and added to 1X APM elution buffer (0.5 M ammonium acetate, 0.5 M DTT, 10 mM EDTA) and tumbled overnight at 4°C. Gel slurry was then loaded onto a pre-wetted 100,000 Da molecular weight cut-off filter (ThermoFisher Scientific) and spun at 14,000 g for 15 min to separate RNA (eluate) from the gel pieces (retentate). Columns were washed with 200 μL of 1X APM elution buffer and spun at 14,000 g for an additional 15 min. Flow-through was collected into a fresh tube. 1 μL of 10 μg/μL glycogen and 1 mL of cold ethanol were added to precipitate the CoA-RNA. RNA was resuspended in nuclease-free water and stored at −20°C until further use.

**Illumina sequencing and data analysis**. Following TURBO DNase treatment and partitioning on APM gels [37] (detailed in Supplemental Methods), purified RNA was reverse transcribed using BST 3.0 DNA polymerase, which we have found reads through structured RNA with significantly less bias than several other common RTs [55]. RT and PCR steps appended Illumina adapters and sequencing indices for multiplexing of the libraries [56]. Sequenced populations were analysed using FASTAptameR 2.0 [57,58] to count and normalize reads (FASTAptameR-Count) and to find the 6 nt index motifs (FASTAptameR-Motif Search) (found in ***SI Appendix*** **Tables S2 & S3**) for all samples to determine counts for D2* and D7*.

## Supplementary Material

CoA RNA SI.pdf

## Data Availability

The authors confirm that the data supporting the findings of this study are available within the article and its supplementary materials.
