# Supplementary material for: Post-transcriptional capping generates coenzyme A-linked RNA
Source: RNA Biol. 2023 Nov 30;21(1):1–12. doi: 10.1080/15476286.2023.2288740 (PMC10761072; doi:10.1080/15476286.2023.2288740)
Supplement: CoA RNA SI.pdf [file KRNB_A_2288740_SM9306.pdf]

Supplemental Information for

Post-transcriptional capping generates Coenzyme A-linked RNA

Krishna Sapkota[1,a,†], Jordyn K. Lucas[2,3,†], Jarrett W. Faulkner[1], Matt F. Lichte[2,3,b], Yan-Lin Guo[4], Donald H. Burke[2,3,5 *], and Faqing Huang[1]*

[1]Department of Chemistry and Biochemistry, and [4] Department of Cell and Molecular Biology, University of Southern Mississippi, Hattiesburg, MS 39406
[2] Department of Biochemistry, [3] Bond Life Sciences Center, and [5] Department of Molecular Microbiology & Immunology, University of Missouri, Columbia, MO 65201
a. Current affiliation for KS: Beam Therapeutics Inc, 238 Main St, Cambridge, MA, 02142
b. Current affiliation for MFL: Associated Regional and University Pathologists, Inc., 500 Chipeta Way, Salt Lake City, UT 84108
†. KS and JKL contributed equally

* Donald H. Burke

Email: BurkeDH@Missouri.edu

https://orcid.org/0000-0001-6513-8391

* Faqing Huang

Email: faqing.huang@usm.edu

https://orcid.org/0000-0002-3985-0980

Solid phase synthesis of BKPP

The synthesis of Biotin-Lys-^{14}C-pantetheine (BKP, bottom left of Scheme S1) was performed by solid phase chemistry at 120 µmol scale. The current state of solid phase chemistry for peptide synthesis has reached its perfection, leading to its simplicity, high speed, excellent coupling yield, and high purity of the final released product. The use of highly reactive agents at large excess allows quantitative reactions on the resin surface within a short period of time at room temperature. The usually tedious chromatographic purification procedure for organic synthesis is unnecessary after each coupling reaction on the resin, since the excess reactants and the reaction biproducts are completed washed out easily. Most of the reactions for BKP synthesis were complete after 30 min of gentle agitation at room temperature, allowing the multiple synthetic steps to be completed in a single day.

Overview. As shown in **Scheme S1**, we started the synthesis by reacting commercially available trityl chloride resin with excess of ethylene diamine in DMF (i). The amino functionalized resin was reacted with HCTU-activated Fmoc-Lys(biotin)-OH in DMF to install a biocytin (biotin-Lys) molecule (ii). Fmoc was deprotected by 20% piperidine in DMF (iii) and the resulting amino group was reacted with HCTU-

activated Fmoc-Cys(stbu)-OH to add cysteine (Cys) (iv). After Fmoc deprotection (v), [^{14}C] sodium acetate was activated by HCTU and coupled to the resin by forming an amide linkage (vi). The S-tertbutyl (stbu) group on cysteine was removed by DTT (vii) and the resulting thiol on the resin was reacted with excess of 1,3-diiodopropane to form a thioether (viii). Pantetheine was produced by reducing commercially available pantethine (oxidized pantetheine dimer via a disulfide bond) with DTT, followed by ether precipitation to remove DTT (not shown), and reacted with the iodo functional group on the beads to form a thioether (ix). The product on the resin was deprotected and released from the beads by TFA (x), precipitated in ether, phosphorylated by recombinantly expressed PanK/CoaA (1) (xi), and purified by HPLC to yield the pPant analog BKPP in high purity.

i)-iii) Resin-immobilized biocytin. Chlorotrityl resin (100 mg, 120 μmol capacity, Chem-Impex International catalog #12996) was swelled in dry DMF for 1 h. The solvent was drained and 1 mL ethylene diamine in dry DMF (1:1 v/v) was added onto the beads. The reaction was gently agitated at room temperature (rt) for 30 min to yield amino-functionalized resin. The solvent was drained, and the beads were washed thrice with 5 mL DMF. N^{α}-Fmoc-N^{ε}-biotinyl-L-lysine, 190 mg (325 μmol), (Chem-Impex International, catalog #04988) was activated by adding 130 mg (315 μmol, 0.96 eq.) of HCTU in 1 mL 20% NMM/DMF. The reaction produced yellow colored solution upon incubation at rt for 5 min. It was added to the beads and agitated for 30 min. The solvent was drained, and the resin was washed thrice with 5 mL DMF. Any unreacted amino group on the beads was capped by adding 100 μl acetic anhydride (1 mmol) in 1 mL DMF to the beads followed by agitation at rt for 30 min. Deprotection of the Fmoc group was carried out by adding 1 mL 20% piperidine in DMF onto the beads for 5 min at rt. The deprotection product was collected by draining the solvent and quantified by UV spectrophotometry at 300 nm with 9400 $cm^{-1}.M^{-1}$, which showed nearly quantitative loading (~98%) of biocytin onto the beads.

iv)-v) Coupling of Fmoc-Cys(Stbu)-OH. The deprotected amino group on resin-linked biocytin was coupled with HCTU-activated carboxyl group of Fmoc-Cys(Stbu)-OH, forming an amide bond. Fmoc-Cys(Stbu)-OH (Chem-Impex International, catalog #02403) (172 mg, 400 μmol) and 157 mg (380 μmol) HCTU were dissolved in 1 mL 20% NMM/DMF and allowed to react at rt for 5 min. The solution was then added to the beads and agitated for 1 h at rt. The solvent was drained, and the resin was washed thrice with 2 mL DMF. Fmoc deprotection was carried out as above. UV quantification showed quantitative yield of the coupling reaction.

vi) Coupling of ^{14}C acetate. The reactive amino group of resin-linked Cys generated after Fmoc deprotection was used to install a ^{14}C-radiolabeled acetate as a reporter tag. Sodium acetate (5 mg, 60 μmol) was dissolved in 0.6 mL water to yield a 0.1 M solution, to which was added 100 μl sodium [1-^{14}C] acetate (Perkin Elmer, NEC084H001MC, 1 mCi/mL, 2 μmol). The resulting solution was frozen and lyophilized to obtain 62 μmol of sodium acetate with 100 μCi total radioactivity. HCTU (25 mg, 60 μmol) and 0.5 mL 20% NMM/DMF were added to the dried sodium ^{14}C-acetate to activate the carboxyl group. The reaction was vortexed at rt for 10 min until completely dissolved. The activated ^{14}C-acetate was added to the beads and allowed to react for 1 h with gentle agitation. Non-radiolabeled sodium acetate, 41 mg, was activated in the same way and added to the beads to cap any unreacted amino groups. The solvent was drained and the radiolabeling yield was found to be ~70% as determined by liquid scintillation counting.

vii) Stbu deprotection. The deprotection of the stbu group was carried out by DTT-mediated disulfide reduction. DTT (77 mg) was dissolved in 0.5 mL DMF and added to the beads. DIPEA (30 μL) was also

added to create a basic environment for the reduction. The reaction was carried out at 60 °C. A small portion of the beads were taken, treated with deprotection reagent (TFA/DCM/TIPS in 1:18:1 v/v) to release product from the beads, and the reaction progress was analyzed by RP-HPLC. HPLC analysis showed that the reaction was completed in 2 h at 60 °C. Solvent was drained and the resin was washed thrice with 4 mL DMF.

viii) Coupling of 1,3-diiodopropane. The thiol group on the beads was treated with 1,3 diiodopropane to yield an iodo-functional group. 1,3-Diiodopropane (Oakwood chemical, catalog #003098) (191 mg, 650 µmol) in 500 µL DMF was added to the beads and reacted at rt for 30 min with gentle agitation. Excess solvent and unreacted 1,3-diiodopropane were drained and the resin were washed thrice with 4 mL DMF. The iodo functional group generated this way was used to react with pantetheine in the next step of synthesis.

ix) Coupling of pantetheine. Pantethine (oxidized pantetheine dimer, Chem-Impex International, catalog #00240) was reduced with DTT to yield pantetheine. Pantethine (150 mg, 270 µmol) was dissolved in 2 mL DMF, followed by adding 63 mg (405 µmol) DTT. The reduction was carried out at rt for 1 h with gentle stirring. The pantetheine product was precipitated by adding 5 mL diethyl ether, washed, and dried in a desiccator. The excess of DTT remained in solution during the precipitation step. It was then dissolved in 1 ml DMF and added to the resin. The reaction was carried out at rt for 30 min with gentle agitation. The solvent was drained, and the resin was washed thrice with 2 mL DMF, completing the solid phase synthesis of BKP.

x) Deprotection from the beads. The product BKP was cleaved from the amino-trityl resin by adding 1 mL cleavage cocktail composed of TFA/DCM/TIPS in 1:18:1 ratio at rt for 10 min. The solution was drained directly into 10 mL ether to precipitate the product. Beads were washed thrice with 0.5 mL dichloromethane (DCM) and drained into diethyl ether. The precipitate was collected by centrifugation and dried at reduced pressure in a desiccator to yield 78 mg (90 µmol, 75% based on 120 µmol starting chlorotrityl resin in step i) of BKP as a white gummy solid, which was used directly to make BKPP by PanK.

xi) Phosphorylation of BKP to form BKPP. Phosphorylation of BKP to form Biotin-Lys-^{14}C-phosphopantetheine (BKPP, bottom right of **Scheme S1**) was carried out by recombinant pantothenate kinase (PanK) purified from *E. coli* (**Fig. S1*A***). BKP (7.8 mg, 9 µmol) was dissolved in 1 mL reaction buffer (50 mM Tris pH 7.0, 10 mM KCl, 1 mM $MgCl_2$) and 11 mg (20 µmol) ATP was added. The reaction was started by adding 0.2 mg PanK and incubated at 37 °C. Reaction progress was analyzed by HPLC and found to be completed in 2 h. Activity of the enzyme in phosphorylating BKP was found to be comparable to its natural substrate pantetheine (not shown). The BKPP product was purified by reverse phase HPLC. The reaction was loaded into a 4.6 x 250 mm C18 column equilibrated with 10% 40 mM KH_2PO_4 at a flow rate of 1 mL/min. The column was washed with 5% acetonitrile for 5 min to remove ATP and ADP. The product was eluted with 50:50 MeCN:Water with no buffer. It was then dried under reduced pressure at 60 °C to yield 7.7 mg of pure BKPP product (8.2 µmol, 90% final yield based on 9 µmol starting BKP). The concentrated sample was further analyzed by liquid scintillation counting and was stored as 5 mM aqueous solution having radioactivity of 50,000 CPM/µL.

Expression and purification of enzymes

Recombinant versions of PanK (CoaA) and PPAT (CoaD), were expressed and purified according to a modified version of our published protocol (1). Briefly, plasmids encoding *coaA* and *coaD* (Addgene # 50386 & 50388) (2, 3) were transformed separately into *E. coli* strain BL21(DE3). Single colonies were isolated and grown in LB/Kan media until OD600 reached 0.6. Protein expression was induced by adding 500 µM IPTG at 37 °C for 4 h. Cells were harvested, lysed by sonication (20s on, 40s rest on ice, 5 cycles), and centrifuged at 40,000g to clear the lysate. Supernatant was loaded into a Ni-NTA resin preequilibrated with 50 mM tris, 300 mM NaCl, and 10 mM Imidazole pH 8.0 and washed extensively, and the bound protein was eluted by the same buffer containing 200 mM imidazole. Membrane filters of 10,000 Da cut-off were used to remove imidazole, and enzyme concentrations were estimated by UV spectroscopy at 280 nm using extinction coefficients of 43,830 and 9,330 $M^{-1}cm^{-1}$ for PanK and PPAT, respectively (4). Purified PanK and PPAT are shown in **Fig. S1*A* & S1*B***, respectively. Glycerol was added to final 50% and proteins were stored in -20 °C until use.

RNA transcripts

DNA templates (**Table S1**) were ordered from Integrated DNA Technologies (IDT). Each RNA was transcribed *in vitro* by mixing 100 pmol of top strand oligo and 100 pmol of bottom strand oligo, using the Y639F T7 RNA polymerase (5), *in vitro* transcription buffer (50 mM Tris-HCl pH 7.5, 15 mM $MgCl_2$, 5 mM DTT, and 2 mM spermidine), and 2 mM each of ATP, UTP, GTP, CTP. Transcription reactions were incubated at 37 °C overnight (approximately 16 h) and terminated by the addition of denaturing gel loading dye (90% formamide, 50 mM EDTA and 0.01% of xylene cyanol and bromophenol blue). Transcripts were subsequently purified by denaturing polyacrylamide gel electrophoresis (5-8% TBE-PAGE, 8 M urea). Transcriptions were also carried out by using high yield transcription kit (Epicentre) following manufacturer's protocol. Bands corresponding to the expected product sizes were visualized by UV shadow, excised from the gel, and eluted by tumbling overnight at 4 °C in 300 mM sodium acetate pH 5.4. Eluates were ethanol precipitated, resuspended in nuclease-free water, and stored at -20°C until further use. A NanoDropOne spectrophotometer (Thermo Fisher Scientific) was used to determine specific RNA concentrations for all assays. Secondary structures were predicted by Mfold (6).

Preparation of RNAI

A template DNA for RNAI transcription was prepared by PCR. Gene-specific primers were used to amplify the 109 bp region of RNAI (including the additional ag or gg added to the 5' end of 107 bp native RNAI) (**Fig. S3**) from the above *PPAT* plasmid (Addgene #50388). The dinucleotide 'AG' (for φ2.5 promoter) or 'GG' (for φ6.5 promoter) were added during PCR to meet the requirements of T7 transcription. The PCR reaction was concentrated to 10x by Zymo DNA clean and concentrator kit following manufacturer's protocol. Transcription was carried out at 37 °C for 3 h. A representative 20 µL transcription contained 2 µL 10x buffer, 2 µL 100 mM DTT, 6 µL NTP mix (25 mM each), 0.5 µL RNase inhibitor, 2 µL 10x template DNA and 2 µL T7 RNA polymerase. Internally ^{32}P labeled RNAI was prepared by transcription under the same condition but including [α-^{32}P]ATP. RNA was purified by RNA Clean and Concentrator kit (Zymo) following manufacturer's protocol, quantified by UV and stored in -20 °C until use.

Preparation of 5 mer RNA

An abortive *in vitro* transcription with modifications was used to prepare 5 mer RNA. A dsDNA template was prepared by annealing a DNA oligo containing T7 φ2.5 promoter and an appropriate sequence to encode 10 mer RNA with its complementary oligo (**Fig. S4*A***). *In vitro* transcription reaction was set to contain 10 μM template DNA and 25 mM ATP and GTP each (UTP and CTP were omitted to prevent run-off synthesis), in addition to the common components mentioned above, [α-^{32}P]ATP (Perkin Elmer) was included to radiolabel RNA internally (**Fig. S5**) Transcriptions produced a mixture of 2 mer, 3 mer, 4 mer and 5 mer RNAs, from which the 5 mer was either gel purified from 20% denaturing PAGE (for radiolabeled 5 mer, **Fig. S5**) or purified by ion-pairing reverse phase HPLC (for non-radiolabeled 5 mer) (**Fig. S4*B***). HPLC conditions were: 4.6 x 250 mm C18 column (Econosphere), flow rate:1 mL/min, solvents: 90% 0.1 M triethylamine-acetate buffer pH 7.0, 8% acetonitrile, and 2% water in isocratic conditions. Peaks were collected, lyophilized, characterized by mass spectrometry, and stored at -20 °C until use. MALDI-ToF in negative ion mode confirmed the identity of the purified 5 mer RNA, which showed a peak having m/z of 1855.5 (expected 1855), along with two other peaks having m/z of 927.2 and 617.9, corresponding to the 5 mer RNA with -2 and -3 charges, respectively (**Fig. S4*C***).

Preparation of 22 mer RNA

The dsDNA templates were prepared by annealing the top strand oligo containing the T7 φ2.5 promoter with the corresponding bottom strand DNA oligos of desired sequences (D2, D3, D4, D5, D7 and D10) with their complementary oligos (**Table S1**). Transcription was carried out at 37 °C for 3 h as above. RNA precipitation was carried out at -20 °C for 1 h by adding NaOAc (0.3 M final) and 3 vol. of EtOH. The pellet was washed with 500μL 70% EtOH and resuspended in nuclease free water, quantified by UV and stored in -20 °C until use.

PPAT-catalyzed reaction kinetics

PPAT catalyzes dpCoA synthesis from ATP and pPant following a random sequential Bi-Bi mechanism (4). Detailed reaction kinetic analysis involving bireactants in both forward and reverse directions is rather complicated and beyond the scope of the current investigation. However, limited kinetic study was conducted to provide useful information regarding four aspects of PPAT activity: 1) the relative rates of reactions of the natural substrate pPant vs its synthetic analog BKPP, 2) the relative rates of reactions of the natural substrate ATP vs ATP-RNA, 3) comparison of different RNA constructs with the same length but varying 5' unstructured regions, and 4) the inhibition of CoA-RNA synthesis in the presence of ATP. This information allows for a meaningful understanding of PPAT-catalyzed CoA-RNA synthesis *in vitro* and that informs discussion on the probability of PPAT-catalyzed CoA-RNA synthesis *in vivo*.

To determine reaction rates, reactions were run at 37 °C in a buffer (20 mM Tris, pH 7.5, 100 mM NaCl, 10 mM KCl, 5 mM $MgCl_2$) containing defined concentrations of PPAT and both substrates. Aliquots were taken out at defined time points up to 4 h and frozen immediately until analysis. Products were separated from reactants by either HPLC for dpCoA synthesis (**Fig. S2*A***) or denaturing PAGE followed by UV and

phosphorimaging quantitation (**Fig. 2*C***). Internally ^{32}P labeled 5 mer RNA was prepared by *in vitro* transcription (**Fig. S5**) and used to study the kinetics of PPAT-catalyzed synthesis of CoA-RNA and BK-CoA-RNA. *In vitro* capping assays were carried out in a PPAT buffer containing 10 µM 5 mer RNA, 500 nM PPAT, and 10-200 µM of pPant or BKPP. Reactions were carried out at 37 °C for the specified time using specified concentrations of pPant. The reactions were quenched by freezing at -20 °C and separated by 20% denaturing PAGE. Gels were dried, exposed to a phosphor storage screen overnight and visualized by phosphorimaging. Band radioactivity was quantified by Volume Analysis of Quantity One software. The initial velocity (V_0) was determined by linear regression of product concentration vs reaction time (**Fig. 2*D***). Since only a few percent of product was formed even at longest time points (4 h), product inhibition was negligible, thereby greatly simplifying kinetic analysis.

Due to the limited kinetic data involving restricted substrate concentrations, V_{max} and k_{cat} could not be obtained directly from substrate saturation plots. Furthermore, since the K_M for ATP is 220 µM, it is reasonable to think that the K_M for ATP-RNA is similar to or higher than 220 µM, making it impractical to directly determine K_M for ATP-RNA. We therefore sought to calculate V_{max} and k_{cat} under different assumptions.

There are different equations to describe the relationship between V_0 and V_{max} for a sequential Bi-Bi mechanism in the literature:

Steady-state ordered sequential Bi-Bi (7):

$$V_0 = V_{max}AB/(K_{ia}K_{mB} + K_{mA}B + K_{mB}A + AB) \quad \textbf{(1)}$$

Rapid equilibrium random sequential Bi-Bi (7):

$$V_0 = V_{max}AB/(\alpha K_A K_B + \alpha K_A B + \alpha K_B A + AB) \quad \textbf{(2)}$$

Or (8):

$$V_0 = V_{max}AB/(K_S{}^A K_m{}^B + K_m{}^B A + K_m{}^A B + AB) \quad \textbf{(3)}$$

However, they are the same formula, if $\alpha K_A = K_m{}^A = K_{mA}$, $\alpha K_B = K_m{}^B = K_{mB}$, and $K_{ia} = K_A = K_S{}^A$ (9). In fact, steady state ordered Bi-Bi and rapid equilibrium random sequential Bi-Bi are indistinguishable in practice (7, 10).

Rearranging Eq (**1**):

$$V_0 = V_{max}/(K_{ia}K_{mB}/AB + K_{mA}/A + K_{mB}/B + 1) \quad \textbf{(4)}$$

At the saturating B and a constant A, the maximum rate V_{satB} may be obtained. V_{max} can then be calculated (after eliminating $K_{ia}K_{mB}/AB$ and K_{mB}/B terms) by:

$$V_{max} = V_{sat}{}^B/(K_{mA}/A + 1) \quad \textbf{(5)}$$

For dpCoA synthesis V_{max} calculation of our purified PPAT, we used above Eq (**1**) with A = B = 200 µM (reaction conditions), V_0 = 0.72 µM/min (**Fig. S2*B***), K_{mA} = 4.7 µM, K_{mA} = 220 µM, and K_{ia} = 1.85 µM (4), yielding V_{max} = 1.54 µM/min. Dividing the value by 50 nM PPAT for the reaction yields k_{cat} = 30.8

min^{-1}, which is about 1/3 of the value reported (4), indicating good quality of PPAT preparation, considering different enzyme preparations, reaction conditions (temperature, pH), and assay methods (HPLC vs coupled enzyme assay).

For PPAT-catalyzed synthesis of CoA-RNA and BK-CoA-RNA, we first plotted the initial reaction velocity obtained from **Fig. 2*D*** against substrate (pPant and BKPP) concentration (**Fig. S6**). We then fitted the data with a simple Michaelis-Menten equation $V_0 = V_{sat}^B/(K_{ap}^B + 1)$, where V_{sat}^B and K_{ap}^B are the maximum reaction rate and apparent Michaelis constant, respectively, for substrate B (pPant and BKPP) under the constant substrate A concentration (10 µM ATP-RNA). The resulting V_{sat}^{Pant} and V_{sat}^{BKPP} are 2.4 and 0.6 nM/min, respectively, and corresponding K_{ap}^{pPant} and K_{ap}^{BKPP} are 14 and 8 µM. Although these values are within 3 times of the reported K_m^{pPant} of 4.7 µM (4), K_{ap}^{Pant} and K_{ap}^{BKPP} are not the same as K_m^{pPant} and K_m^{BKPP}(under saturating BKPP and pPant, respectively), since K_{ap}^{Pant} and K_{ap}^{BKPP} were not derived from saturating ATP-RNA concentration.

Although V_{max} can be calculated from Eq (**5**), it is impractical to determine $K_m^{ATP\text{-}RNA}$. If assuming the similar value of 220 µM for K_m^{ATP} (4), we can estimate V_{max} for PPAT-catalyzed pPant/ATP-RNA and BKPP/ATP-RNA reactions using 10 µM ATP-RNA by Eq (**5**):

$$V_{max} = V_{sat}^B/(220/10 + 1) = 23V_{sat}^B, \text{ or 55 and 13 nM/min}$$

$k_{cat} = V_{max}$ /[PPAT], yielding 0.11 and 0.026 min^{-1} for CoA-RNA and BK-CoA-RNA synthesis. Therefore, if assuming similar K_M values for analogous substrates for PPAT-catalyzed synthesis of dpCoA and CoA-RNA, dpCoA synthesis is 280 times (30.8 divided by 0.11) more efficient than CoA-RNA synthesis under the same conditions.

Nitrocellulose filter binding assays (Fig. 3*C*, 3*E*, 3*F*)

Radiolabeled RNA was quantified using liquid scintillation countering. Each binding assay sample was at least 30,000 DPM RNA. RNA was incubated with no PPAT to determine background binding of RNA to the nitrocellulose filter and an unfiltered 'no wash' sample was measured to determine the total input signal. To decrease non-specific nucleic acid binding, filters were incubated in 0.5 M KOH for 20 min, washed with MilliQ water, and incubated in 1X binding buffer for 45 min prior to use (11). Trace amounts of radiolabeled and refolded RNA was incubated with varying concentrations of PPAT in 1X binding buffer (20 mM Tris pH 7.5, 100 mM NaCl, 10 mM KCl, 1 mM $MgCl_2$) at 37 °C for 15 min. RNA:PPAT complexes were partitioned from unbound RNA by filtering through a pre-wet, KOH-treated filters under vacuum and immediately washing with 1 mL of 1X binding buffer. Three replicates were performed for each binding assay. Radioactive RNA bound to PPAT on the filters was counted by liquid scintillation counting in 3 mL Emulsifier-safe liquid scintillation fluid (Perkin Elmer, Waltham, MA).

RNA expression plasmids

Plasmid pJKL1 (VectorBuilder, Chicago, Illinois) (**Fig. S8**) was designed for inducible expression of PPAT and constitutive expression of D2* and D7* RNA transcripts (**Fig. 3*D***). Plasmid sequence was confirmed by Sanger Sequencing (University of Missouri DNA Core Facility). Purified plasmid was transformed

into BL21(DE3)pLysS chemically competent cells and colonies were grown on Ampicillin (50 μg/mL) agar plates for 16 h in a 37 °C incubator shaking at 250 rpm. Single colonies were inoculated into 5 mL of 2XYT Ampicillin media and grown at 37 °C for 16 h. 1 mL overnight culture was added to 4 different 50 mL Falcon Tubes containing 25 mL of 2XYT Ampicillin media (two biological replicate each for 'induced' and 'uninduced' cultures). These were incubated in a 37 °C incubator with shaking at 250 rpm. After the cultures reached an OD_{600} of 0.6, IPTG was added to a final concentration of 1 mM to two of the four cultures to induce PPAT expression and incubated at 37 °C for an additional hour. The other two cultures remained uninduced but were incubated at 37 °C for the same amount of time. OD_{600} was measured hourly and all cultures were put on ice when OD_{600} reached 1.45-1.6.

TURBO DNase treatment

Samples of isolated RNA with nucleic acid concentrations greater than 20 μg/mL were diluted to 10 μg/50 μL. 5 μL of sample were set aside after 0, 1, and 2 DNase treatments to be used for PCR as template (without reverse transcription) to evaluate carryover DNA contamination. TURBO DNase (ThermoFisher Scientific) reactions with up to 10 μg of nucleic acid were assembled in 1X TURBO DNase buffer with 2 U of TURBO DNase and incubated at 37 °C for 30 min, then 5 μL of TURBO DNase Inactivation Reagent (ThermoFisher Scientific) was added and incubated at room temperature for 5 min with intermittent mixing to keep the reagent in solution. Samples were spun on a tabletop centrifuge for ~90 sec to separate tDNase-treated samples (supernatant) and inactivation reagent. The supernatant was collected and concentrated by ethanol precipitation. PCR to test the effectiveness of TURBO DNase treatments used 16S rRNA primers.

APM gels & elution

[(N-Acryloylamino) Phenyl] Mercuric Chloride (APM) stock solution was made as previously described (12). To pour a tri-layer APM gel, the first layer of polyacrylamide (about 20 mL) was poured in an upright gel casing. 1 mL of MilliQ water was added directly after pouring the bottom layer to create a smooth interface between layers. After allowing the first layer to polymerize for approximately 30 min, water was removed and the second, APM-containing layer was added, consisting of 1 mL of polyacrylamide, 1 μL TEMED, 10 μL 10% ammonium persulfate (APS), and 200 μL of APM stock solution. The APM layer was covered by a fresh 1 mL layer of water and allowed to polymerize for approximately 30 min. Excess water was then removed and the final 10 mL layer of polyacrylamide was added along with the comb for generating wells and allowed to polymerize for 30 min.

Illumina sequencing and data analysis

Following TURBO DNase treatment and partitioning on APM gels (12), purified RNA was reverse transcribed using BST 3.0 DNA polymerase, which we have found reads through structured RNA with significantly less bias than several other common RTs (Lucas, Gruenke & Burke, 2023 *RNA* in press). Reactions included a specific reverse primer that amplifies both D2* and D7* RNA templates, 1X

isothermal buffer (New England Biolabs), 6 mM $MgSO_4$, 1.6 mM dNTP mix, and 16 U BST enzyme in a 25 μL reaction volume and incubated at 72 °C for 1 h. The primers for reverse transcription and PCR anneal to the same sequences on D2* and D7* (**Table S1**). RT and PCR steps appended Illumina adapters and sequencing indices for multiplexing of the libraries as previously described (13). Sequencing was performed on an Illumina NextSeq 500 (University of Missouri Genomics Technology Core). Although paired-end reads generated Read 1 and Read 2 for each selection round, a single 300 nt read provided enough coverage such that no additional information was gained through read pairing. Populations were demultiplexed, and the relevant sequence information was found and used from Read 1 (5′ HTS primer binding sequence, 6 nt index, 3′ HTS primer binding sequence), and all data shown represent reads from Read 1 only. Data preprocessing was performed using cutadapt (14) to trim 5′ and 3′ PBS and to discard any uncut sequences or sequences with lengths not within ± 3 nt of the expected size (26 nt) after trimming (**Table S2 & S3**). These populations were then analyzed using FASTAptameR 2.0 (15, 16) to count and normalize reads (FASTAptameR-Count) and to find the 6 nt index motifs (FASTAptameR-Motif Search) (found in **Table S2 & S3**) for all samples to determine counts for D2* and D7*.

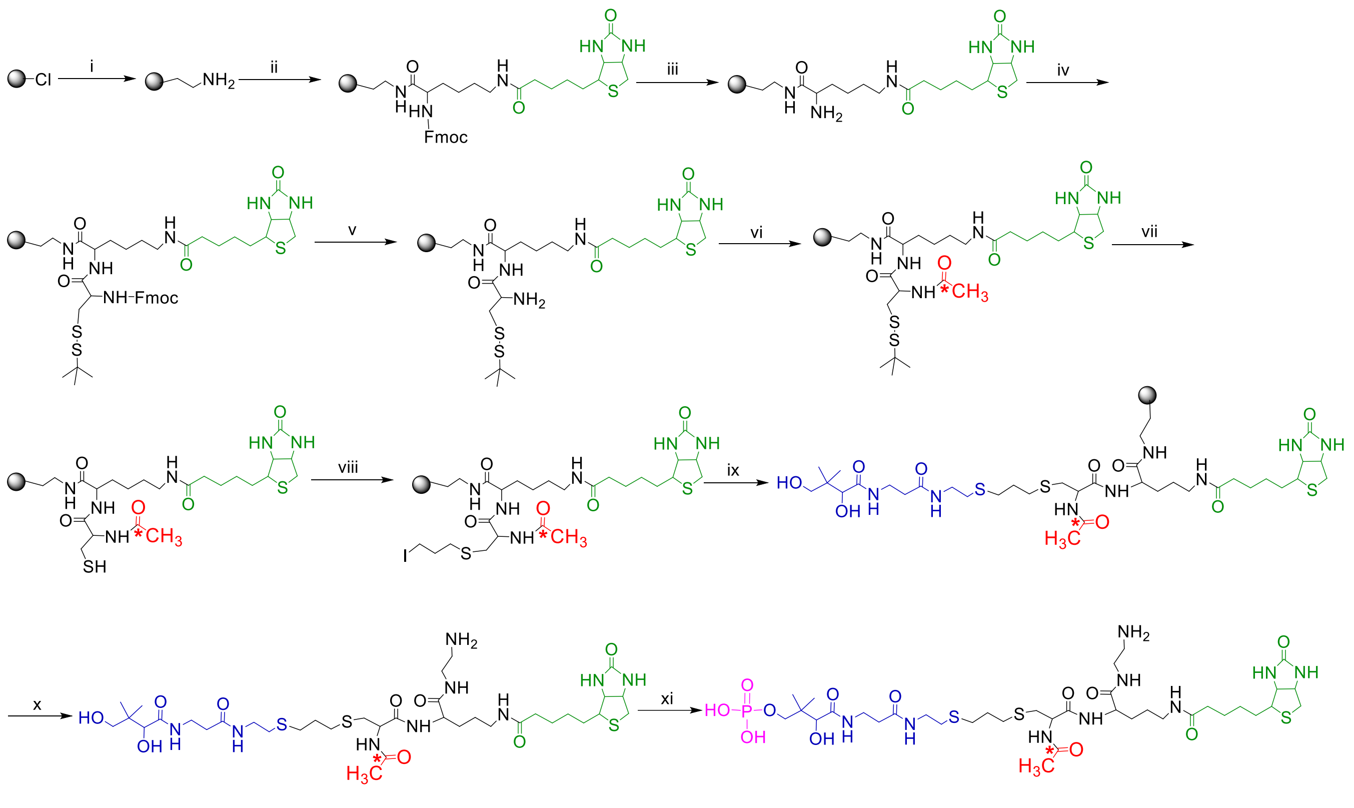

Biotin-Lys-^{14}C-pantetheine (BKP) **Biotin-Lys-^{14}C-phosphopantetheine (BKPP)**

Scheme S1. Synthesis of Biotin-Lys-^{14}C-phosphopantetheine (BKPP). Phosphopantetheine analog BKPP was synthesized by multistep solid phase synthesis. Reagents and conditions: (i) ethylene diamine, DMF, 10 min, rt; (ii) N^{α}-Fmoc-N^{ε}-biotinyl-L-lysine, HCTU, NMM/DMF, 10-30 min, rt; (iii) and (v) 20% piperidine in DMF, rt, 5 min; (iv) Fmoc-Cys(stbu)-OH, HCTU NMM/DMF, 10-30 min, rt; (vi) [1-^{14}C] NaOAc, HCTU, DMF, 1 h, rt; (vii) 1 M DTT, 60 °C, 2 h; (viii) 1,3-diiodopropane, DMF, RT, 30 min, rt; (ix) pantetheine, DMF, rt, 30 min; (x) TFA/DCM/TIPS in 1:18:1, rt, 10 min; (xi) PanK, ATP, 37 °C, 2 h. Abbreviations: DMF – N,N-dimethylformamide; Fmoc – Fluorenylmethyloxycarbonyl; HCTU – O-(1H-6-Chlorobenzotriazole-1-yl)-1,1,3,3-tetramethyluronium; stbu – S-tert butyl; TFA – trifluoroacetic acid; DTT – dithiothreitol; NMM – N-methylmorpholine.

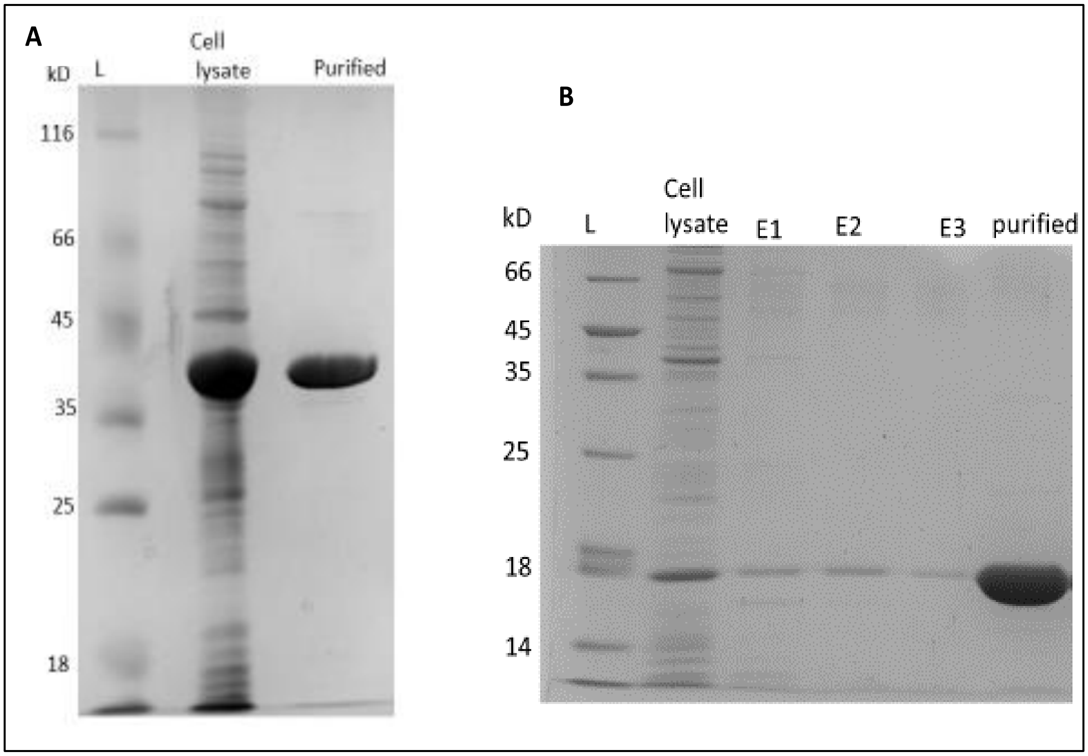

Fig. S1. Expression and purification of recombinant PanK and PPAT. (*A*) PanK was expressed in BL21(DE3) cells as His-tagged recombinant protein and purified by Ni-NTA chromatography. A dark single band in lane 3 shows the good purity of the PanK after purification. (*B*) The PPAT enzyme was purified same way as PanK. A dark band below 18,000 Da marker corresponds to PPAT. After eluting with 200 mM imidazole, both enzymes were concentrated by a membrane filter of 10,000 Da cutoff and stored at -20 °C in 1x storage buffer containing 50% glycerol.

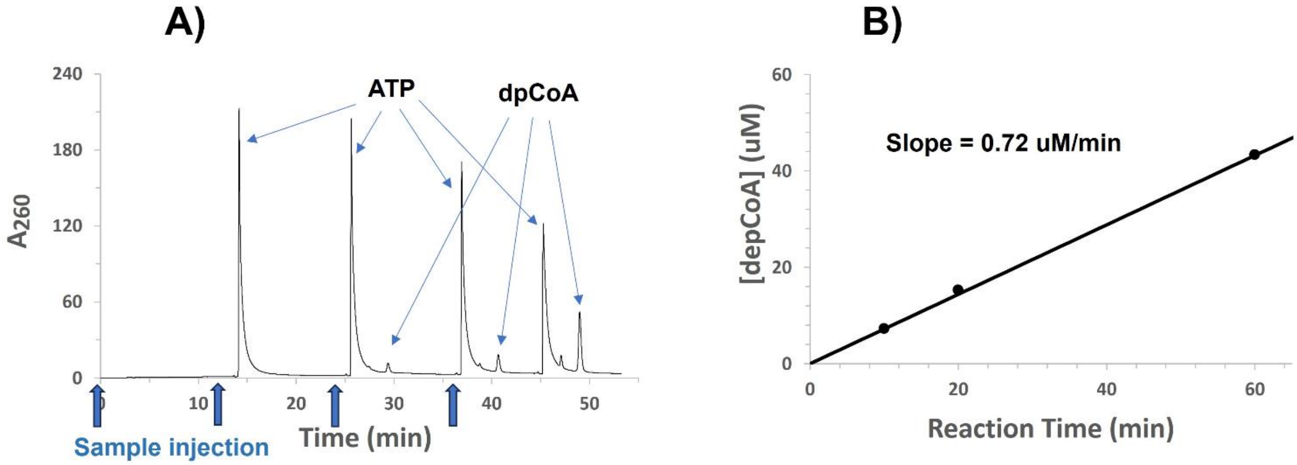

Fig. S2: Kinetic analysis of PPAT-catalyzed pPant reaction with ATP to form dpCoA: (*A*) Four sample injections onto HPLC were performed at an interval of 12 min (bold solid arrows), resulting in 4 sets of peaks of the samples collected at different reaction time (0, 10, 20, 60 min). The reaction was started by incubating 50 nM PPAT with 200 μM ATP and 200 μM pPant in the reaction buffer (20 mM Tris, pH 7.5, 100 mM NaCl, 10 mM KCl, 5 mM $MgCl_2$) at 37 °C. Small aliquots were taken at 0, 10, 20 and 60 min. The reaction was quenched by immediately freezing the samples at -20 °C and analyzed by RP-HPLC. HPLC conditions were: flow rate 1 mL/min, isocratic elution at 42% water, 8% MeCN, 50% 1M TEA-acetate buffer pH 7.0, UV detection at 260 nm. ATP and dpCoA (product) are indicated by green and red arrows, respectively; (*B*) Product dpCoA concentration-time relationship.

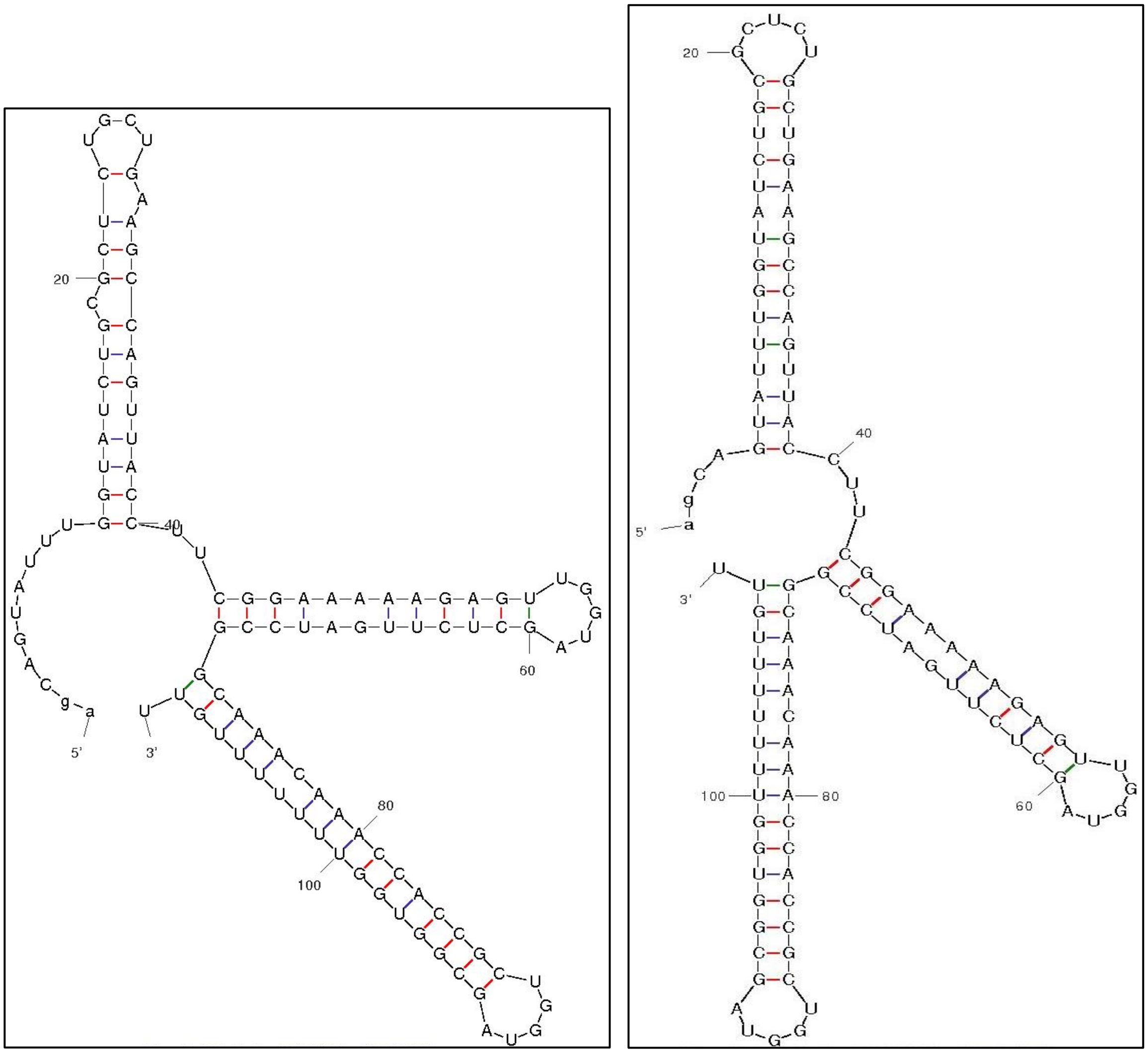

Fig. S3. Predicted secondary structures of *E. coli* RNAI. Mfold server was used to predict the secondary structures that showed two possible structures of comparable free energy (ΔG = -43.8 and 43.1 kcal/mol) with respective 10 and 4 unpaired nucleotides at the 5' terminus. Lower case ag (shown) or gg (not shown) dinucleotides are appended to the 5' ends to aid *in vitro* transcription with T7 RNA Polymerase.

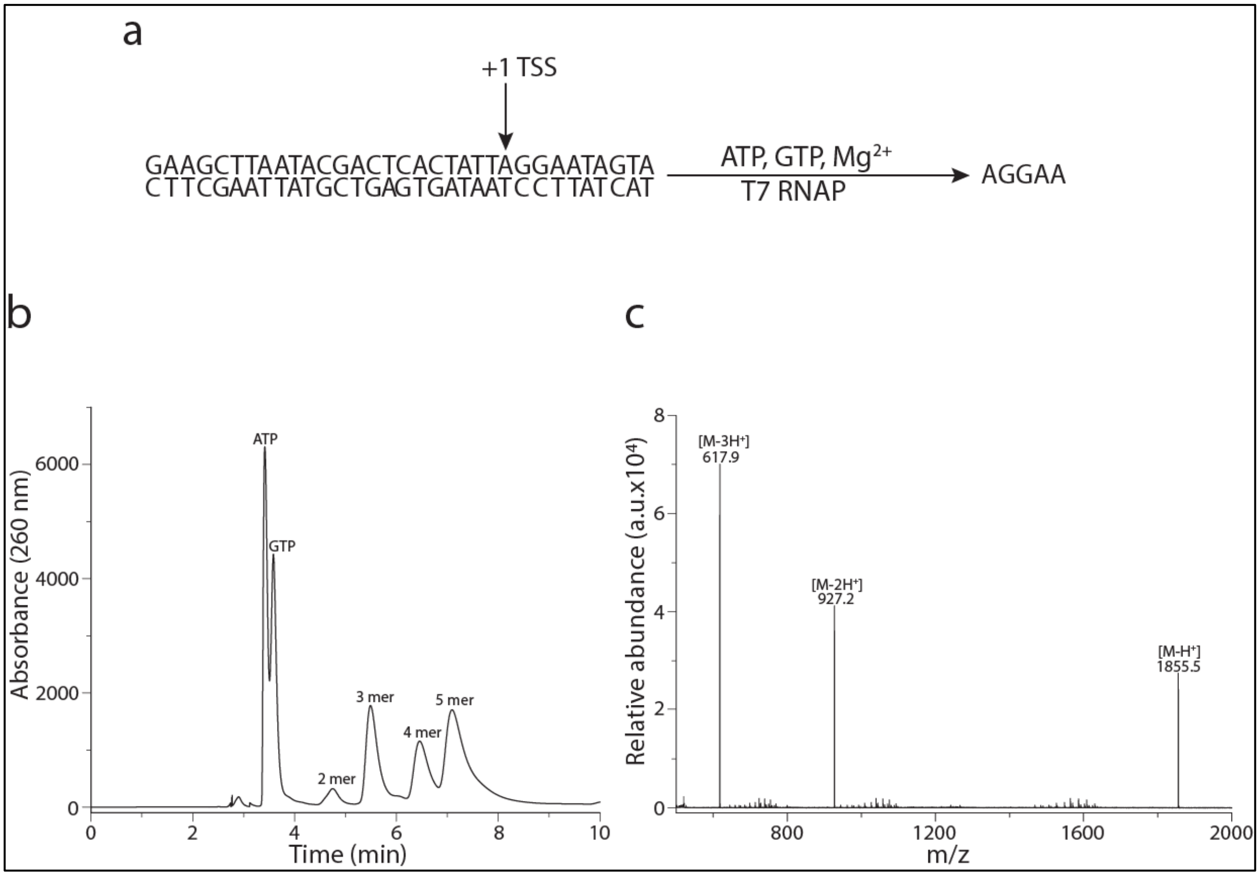

Fig. S4. Abortive *in vitro* transcription was used to prepare 5mer RNA. (*A*) Schematic of transcription template and T7 φ2.5 promoter. Note that because ATP and GTP were the only NTPs in the reaction, transcription terminates after synthesizing the 5mer. (*B*) Ion pairing HPLC was used to obtain pure 5mer RNA. HPLC conditions were: 4.6 x 250 mm C18 column (Econosphere), flow rate:1 mL/min, solvents: 90% 0.1 M triethylamine-acetate buffer pH 7.0, 8% acetonitrile, and 2% water in isocratic conditions. (*C*) The HPLC peak corresponding to the 5 mer from panel (*B*) was lyophilized, and its identity was confirmed by mass spectrometry.

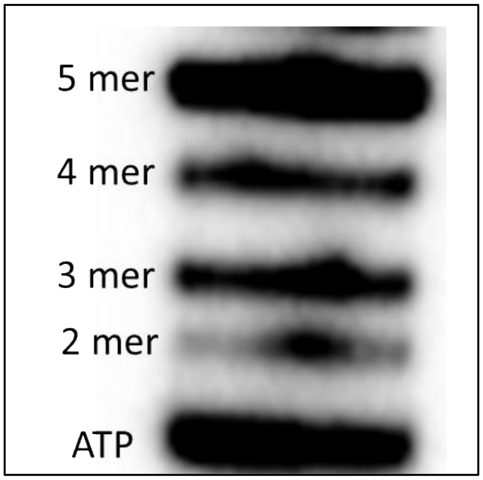

Fig. S5. Purification of radiolabeled 5mer RNA by denaturing PAGE. RNA was prepared by standard *in vitro* transcription using a synthetic DNA template (**Fig. S4*A***). Only two (ATP and GTP) of the four nucleotides were used in the reaction to abort it after 5 nucleotides. Transcription produced a heterogenous mixture of 2 mer, 3 mer, 4 mer and 5 mer RNAs, which were separated on 20% denaturing PAGE of 0.4 mm thickness. The band corresponding to 5 mer RNA was excised and purified by crush and soak method.

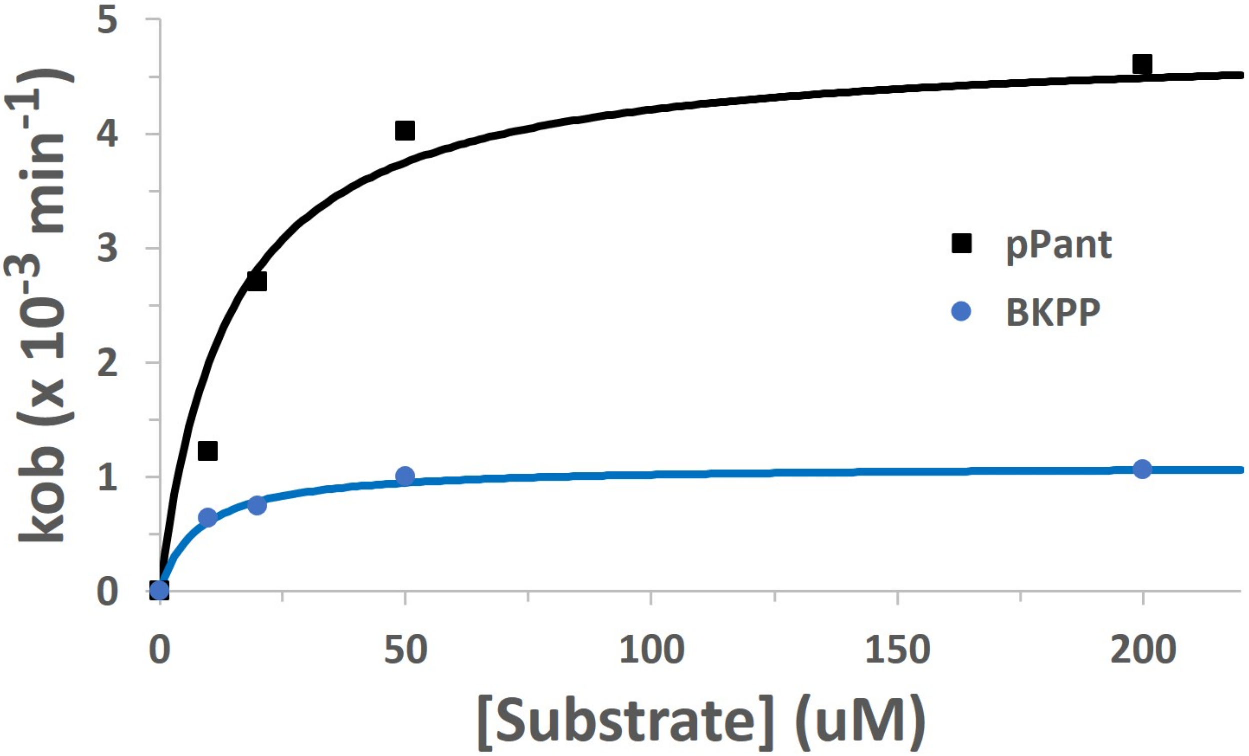

Fig. S6: Michaelis-Menten plotting of initial velocity V_0 (from **Fig. 2*D***) vs substrate (pPant and BKPP) concentration under constant 10 μM [ATP-RNA] and 500 nM PPAT in the reaction buffer (20 mM Tris, pH 7.5, 100 mM NaCl, 10 mM KCl, 5 mM $MgCl_2$) at 37 °C. The maximum rates are 2.4 and 0.6 nM/min for pPant and BKPP, respectively.

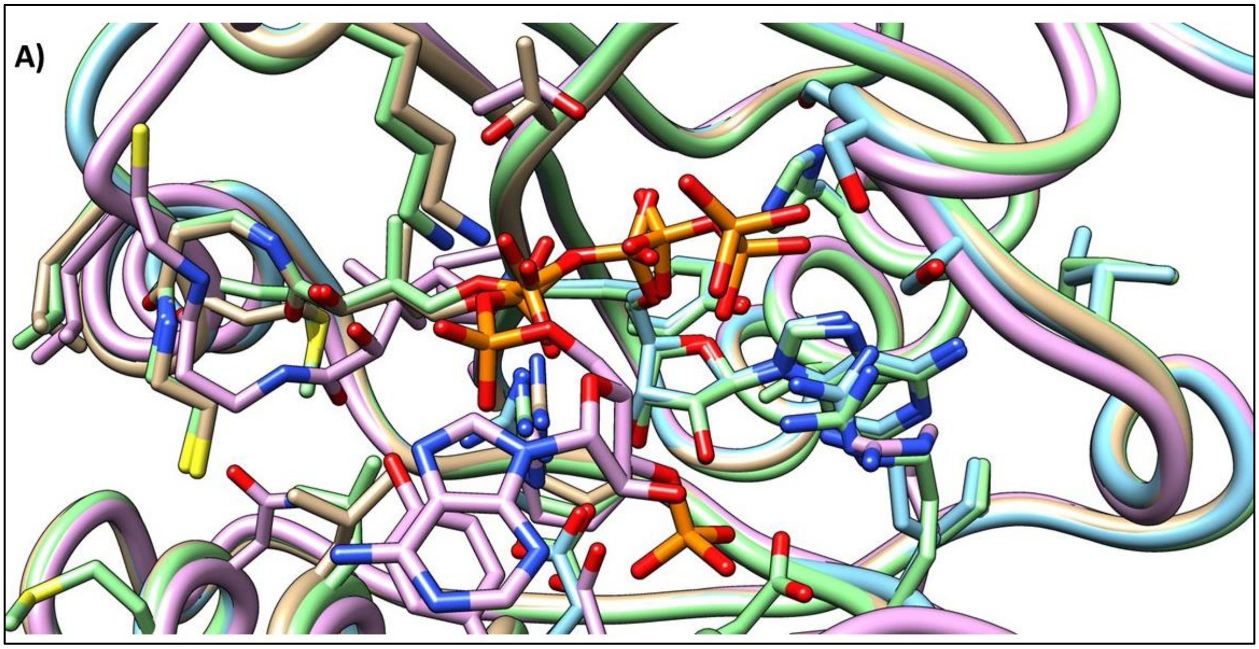
A)

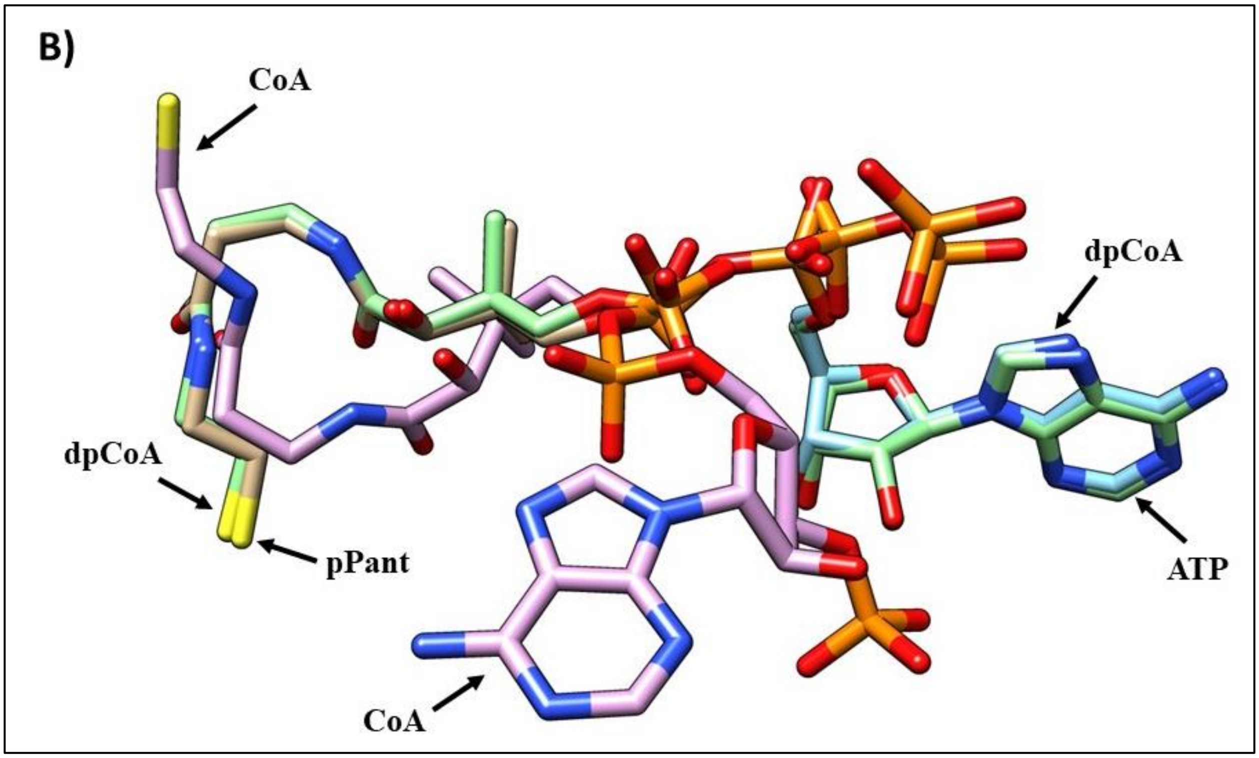
B)
CoA
dpCoA
pPant
dpCoA
ATP
CoA

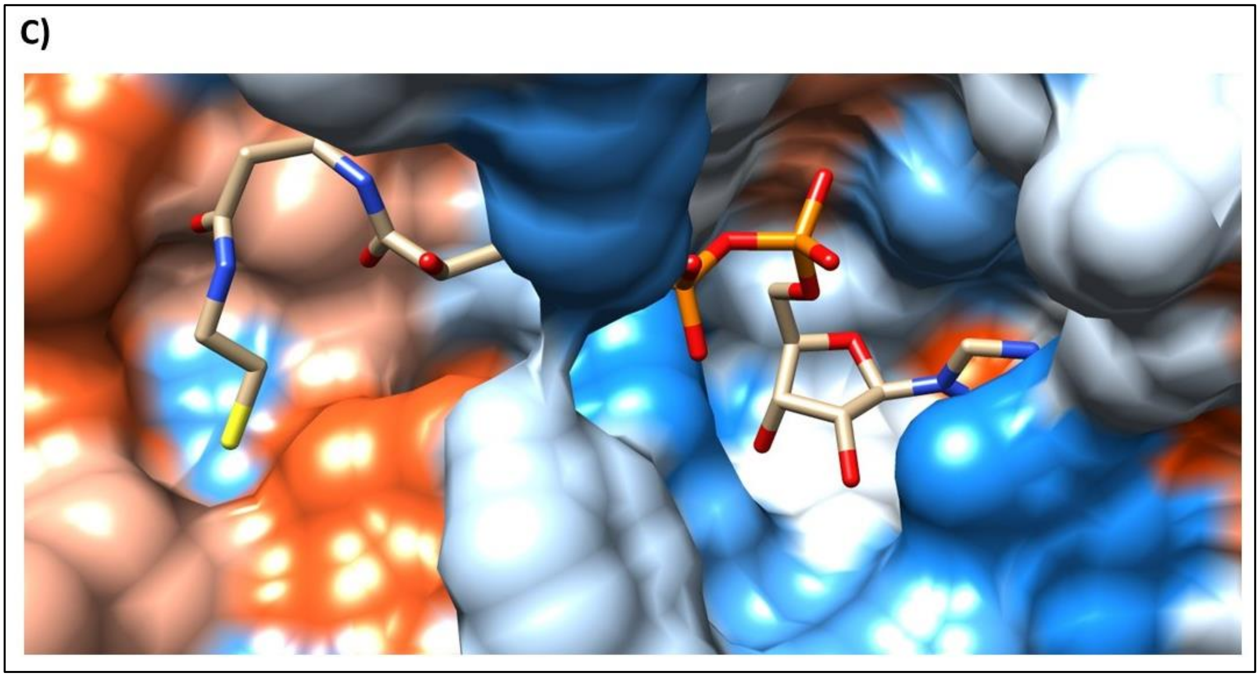

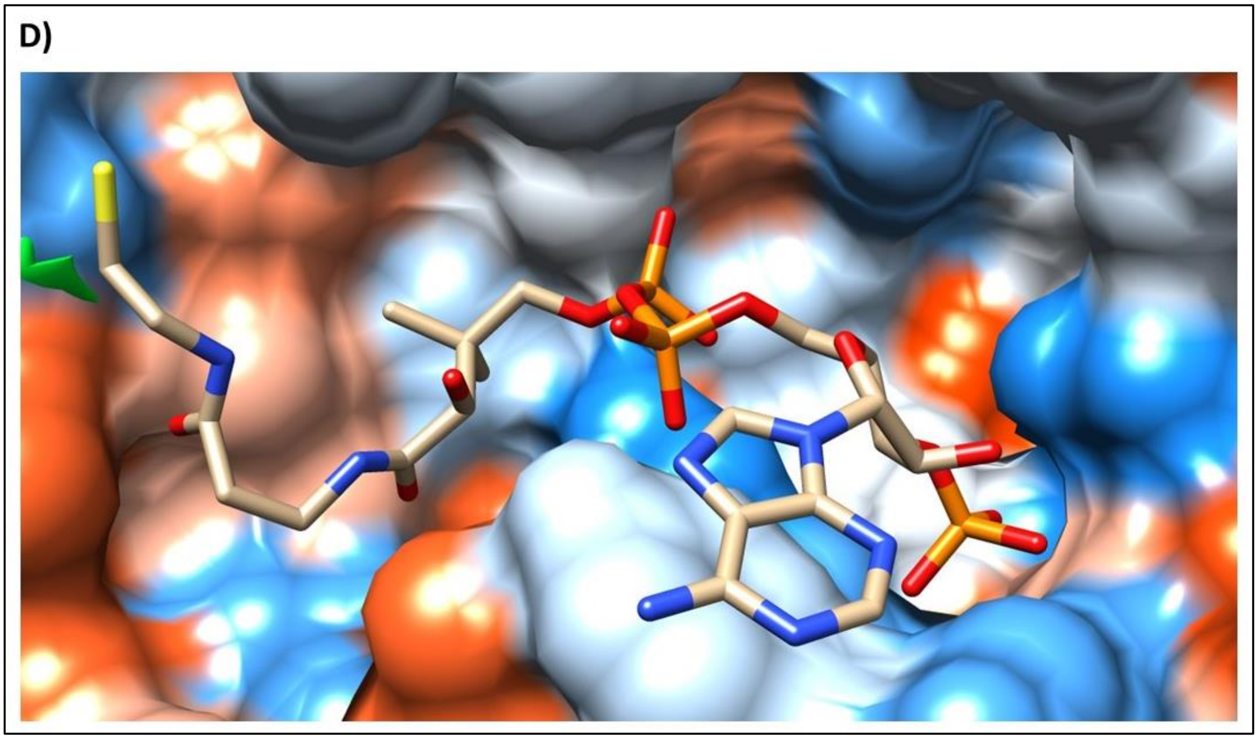

Fig. S7. (*A*) Overlay of the substrate/product binding site of PPAT using UCSF Chimera's MatchMaker function. The whole structures including protein and substrates were included in the alignment. Structures were derived from the following PDB IDs: **1GN8** (PPAT/ATP), **1QJC** (PPAT/pPan), 1B6T (PPAT/dpCoA), and **1H1T** (PPAT/CoA). (*B*) Spatial relationships among substrates and products

derived from the same structure as in (*A*) after removing all protein structures. (*C*) dpCoA binding at the active site. (*D*) CoA binding at a different site. Based on the structures, dpCoA and pPant/ATP occupy nearly identical space with the same orientation at the reaction active site. However, there is no space for a phosphate group at the 3’ ribose of ATP/dpCoA, forcing CoA to occupy a different site with the opposite Pant orientation from that of pPant/dpCoA. Although the CoA binding site can accommodate a 3’ phosphate group, there is no space in this structure for additional chemical groups such as RNA.

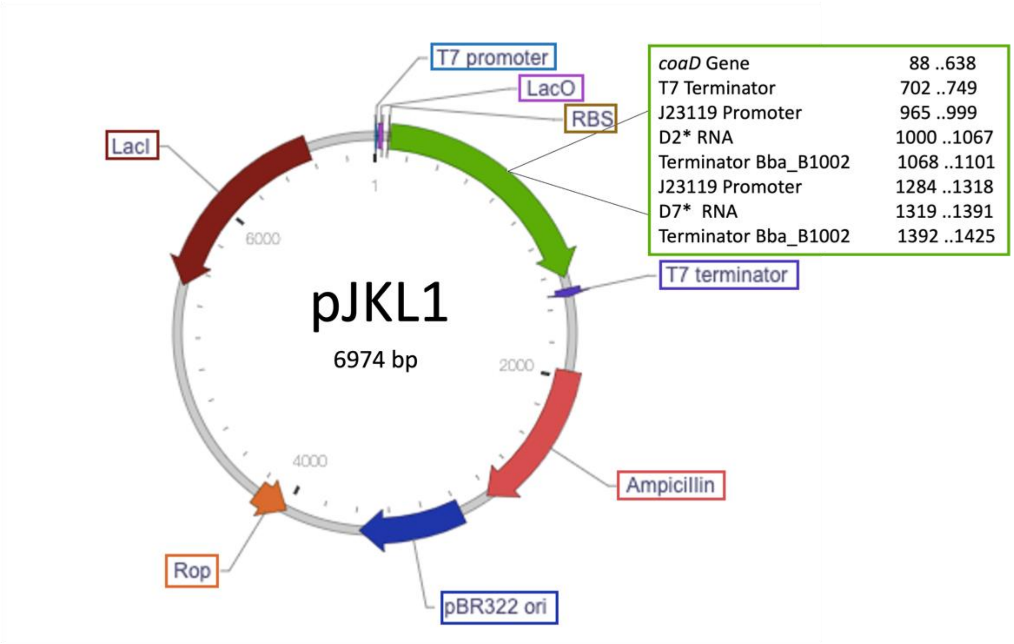

T7 Promoter (1-17)

LacO (20-44)

RBS (76-80)

Insert Region (88-1425)

CoaD Gene (88-638)

T7 Terminator (702-749)

J23119 Promoter (965-999)

D2* RNA (1000-1067)

Terminator Bba_B1002 (1068-1101)

J23119 Promoter (1284-1318)

D7* RNA (1319-1391)

Terminator Bba_B1002 (1392-1425)

T7 Terminator (1490-1536)

Ampicillin (1957-2817)

pBR322 Ori (2988-3576)

Rop (4003-4194)

LacI (5506-6588)

Plasmid Sequence

TAATACGACTCACTATAGGGGAATTGTGAGCGGATAACAATTCCCCTCTAGAAATAATTTTGTTTAACTTTAAGAAGGAGATATA
CCATGCAAAAACGGGCGATTTATCCGGGTACTTTCGATCCCATTACCAATGGTCATATCGATATCGTGACGCGCGCCACGCAGAT
GTTCGATCACGTTATTCTGGCGATTGCCGCCAGCCCCAGTAAAAAACCGATGTTTACCCTGGAAGAGCGTGTGGCACTGGCAC
AGCAGGCAACCGCGCATCTGGGGAACGTGGAAGTGGTCGGGTTTAGTGATTTAATGGCGAACTTCGCCCGTAATCAACACGC
TACGGTGCTGATTCGTGGCCTGCGTGCGGTGGCAGATTTTGAATATGAAATGCAGCTGGCGCACATGAATCGCCACTTAATGCC
GGAACTGGAAAGTGTGTTTCTGATGCCGTCGAAAGAGTGGTCGTTTATCTCTTCATCGTTGGTGAAAGAGGTGGCGCGCCATC
AGGGCGATGTCACCCATTTCCTGCCGGAGAATGTCCATCAGGCGCTGATGGCGAAGTTAGCGTAGCGTTGGATCCGAATTCGA
GCTCCGTCGACAAGCTTGCGGCCGCACTCGAGCACCACCACCACCACCACTGAGATCCGGCTGCTAACAAAGCCCGAAAGGA
AGCTGAGTTGGCTGCTGCCACCGCTGAGCAATAACTAGCATAACCCCTTGGGGCCTCTAAACGGGTCTTGAGGGGTTTTTTGC
AGGTGGCACTTTTCGGGGAAATGTGCGCGGAACCCCTATTTGTTTATTTTTCTAAATACATTCAAATATGTATCCGCTCATGAATT
AATTCGTAACTGCTGCTGGGATTACACATGGCATGGATGAGCTCTACAAATAATGAAACGAATTCAAGCTTGATATCATTCAGGA
CGAGCCTCAGACTCCAGCGTAACTGGACTGCAATCAACTCACTTTGACAGCTAGCTCAGTCCTAGGTATAATGCTAGC**AGGTGC
AGACGATTCGTCACACGTCCTGCACACGGCTGGAAACTACTCATCATCATCGAGCTGGCAGT***CGCAAAAAACCCCGCTTCGG
CGGGGTTTTTTCGC*GTAACTGCTGCTGGGATTACACATGGCATGGATGAGCTCTACAAAGGTCAATACACTACATGGCGTGATT
TCATATGCGCGATTGCTGATCCCCATGTGTATCACTGGCAAACTGTGATGGACGACACCGTCAGTGCGTCCGTCGCGCAGGCTC
TCGATGAGCTGATGCTTTGGGCCGAGGATTGACAGCTAGCTCAGTCCTAGGTATAATGCTAGC**AGGACCAGTGCAGACGATTC
GTCACACGTCCTGCACACGGCTGGAAACACGTAATCATCATCGAGCTGGCAGT***CGCAAAAAACCCCGCTTCGGCGGGGTTTT
TTCGC*GGATCCGCTGCTAACAAAGCCCGAAAGGAAGCTGAGTTGGCTGCTGCCACCGCTGAGCAATAACTAGCATAACCCCTT
GGGGCCTCTAAACGGGTCTTGAGGGGTTTTTTGCTGAAAGGAGGAACTATATCCGGATATCCCGCAAGAGGCCCGGCAGTAC
CGGCATAACCAAGCCTATGCCTACAGCATCCAGGGTGACGGTGCCGAGGATGACGATGAGCGCATTGTTAGATTTCATACACG
GTGCCTGACTGCGTTAGCAATTTAACTGTGATAAACTACCGCATTAAAGCTTATCGATGATAAGCTGTCAAACATGAGAATTCTT
GAAGACGAAAGGGCCTCGTGATACGCCTATTTTTATAGGTTAATGTCATGATAATAATGGTTTCTTAGACGTCAGGTGGCACTTT
TCGGGGAAATGTGCGCGGAACCCCTATTTGTTTATTTTTCTAAATACATTCAAATATGTATCCGCTCATGAGACAATAACCCTGAT
AAATGCTTCAATAATATTGAAAAAGGAAGAGTATGAGTATTCAACATTTCCGTGTCGCCCTTATTCCCTTTTTTGCGGCATTTTGC
CTTCCTGTTTTTGCTCACCCAGAAACGCTGGTGAAAGTAAAAGATGCTGAAGATCAGTTGGGTGCACGAGTGGGTTACATCGA
ACTGGATCTCAACAGCGGTAAGATCCTTGAGAGTTTTCGCCCCGAAGAACGTTTTCCAATGATGAGCACTTTTAAAGTTCTGCT
ATGTGGCGCGGTATTATCCCGTGTTGACGCCGGGCAAGAGCAACTCGGTCGCCGCATACACTATTCTCAGAATGACTTGGTTG
AGTACTCACCAGTCACAGAAAAGCATCTTACGGATGGCATGACAGTAAGAGAATTATGCAGTGCTGCCATAACCATGAGTGATA
ACACTGCGGCCAACTTACTTCTGACAACGATCGGAGGACCGAAGGAGCTAACCGCTTTTTTGCACAACATGGGGGATCATGTA
ACTCGCCTTGATCGTTGGGAACCGGAGCTGAATGAAGCCATACCAAACGACGAGCGTGACACCACGATGCCTGCAGCAATGG
CAACAACGTTGCGCAAACTATTAACTGGCGAACTACTTACTCTAGCTTCCCGGCAACAATTAATAGACTGGATGGAGGCGGATA
AAGTTGCAGGACCACTTCTGCGCTCGGCCCTTCCGGCTGGCTGGTTTATTGCTGATAAATCTGGAGCCGGTGAGCGTGGGTCA
CGCGGTATCATTGCAGCACTGGGGCCAGATGGTAAGCCCTCCCGTATCGTAGTTATCTACACGACGGGGAGTCAGGCAACTAT
GGATGAACGAAATAGACAGATCGCTGAGATAGGTGCCTCACTGATTAAGCATTGGTAACTGTCAGACCAAGTTTACTCATATAT

ACTTTAGATTGATTTAAAACTTCATTTTTAATTTAAAAGGATCTAGGTGAAGATCCTTTTTGATAATCTCATGACCAAAATCCCTTA
ACGTGAGTTTTCGTTCCACTGAGCGTCAGACCCCGTAGAAAAGATCAAAGGATCTTCTTGAGATCCTTTTTTTCTGCGCGTAAT
CTGCTGCTTGCAAACAAAAAAACCACCGCTACCAGCGGTGGTTTGTTTGCCGGATCAAGAGCTACCAACTCTTTTTCCGAAGG
TAACTGGCTTCAGCAGAGCGCAGATACCAAATACTGTCCTTCTAGTGTAGCCGTAGTTAGGCCACCACTTCAAGAACTCTGTAG
CACCGCCTACATACCTCGCTCTGCTAATCCTGTTACCAGTGGCTGCTGCCAGTGGCGATAAGTCGTGTCTTACCGGGTTGGACT
CAAGACGATAGTTACCGGATAAGGCGCAGCGGTCGGGCTGAACGGGGGGTTCGTGCACACAGCCCAGCTTGGAGCGAACG
ACCTACACCGAACTGAGATACCTACAGCGTGAGCTATGAGAAAGCGCCACGCTTCCCGAAGGGAGAAAGGCGGACAGGTATC
CGGTAAGCGGCAGGGTCGGAACAGGAGAGCGCACGAGGGAGCTTCCAGGGGGAAACGCCTGGTATCTTTATAGTCCTGTCG
GGTTTCGCCACCTCTGACTTGAGCGTCGATTTTTGTGATGCTCGTCAGGGGGGCGGAGCCTATGGAAAAACGCCAGCAACGC
GGCCTTTTTACGGTTCCTGGCCTTTTGCTGGCCTTTTGCTCACATGTTCTTTCCTGCGTTATCCCCTGATTCTGTGGATAACCGTA
TTACCGCCTTTGAGTGAGCTGATACCGCTCGCCGCAGCCGAACGACCGAGCGCAGCGAGTCAGTGAGCGAGGAAGCGGAAG
GGCGCCTGATGCGGTATTTTCTCCTTACGCATCTGTGCGGTATTTCACACCGCAATGGTGCACTCTCAGTACAATCTGCTCTGAT
GCCGCATAGTTAAGCCAGTATACACTCCGCTATCGCTACGTGACTGGGTCATGGCTGCGCCCCGACACCCGCCAACACCCGCTG
ACGCGCCCTGACGGGCTTGTCTGCTCCCGGCATCCGCTTACAGACAAGCTGTGACCGTCTCCGGGAGCTGCATGTGTCAGAG
GTTTTCACCGTCATCACCGAAACGCGCGAGGCAGCTGCGGTAAAGCTCATCAGCGTGGTCGTGAAGCGATTCACAGATGTCT
GCCTGTTCATCCGCGTCCAGCTCGTTGAGTTTCTCCAGAAGCGTTAATGTCTGGCTTCTGATAAAGCGGGCCATGTTAAGGGC
GGTTTTTTCCTGTTTGGTCACTGATGCCTCCGTGTAAGGGGGATTTCTGTTCATGGGGGTAATGATACCGATGAAACGAGAGA
GGATGCTCACGATACGGGTTACTGATGATGAACATGCCCGGTTACTGGAACGTTGTGAGGGTAAACAACTGGCGGTATGGATG
CGGCGGGACCAGAGAAAAATCACTCAGGGTCAATGCCAGCGCTTCGTTAATACAGATGTAGGTGTTCCACAGGGTAGCCAGC
AGCATCCTGCGATGCAGATCCGGAACATAATGGTGCAGGGCGCTGACTTCCGCGTTTCCAGACTTTACGAAACACGGAAACC
GAAGACCATTCATGTTGTTGCTCAGGTCGCAGACGTTTTGCAGCAGCAGTCGCTTCACGTTCGCTCGCGTATCGGTGATTCATT
CTGCTAACCAGTAAGGCAACCCCGCCAGCCTAGCCGGGTCCTCAACGACAGGAGCACGATCATGCGCACCCGTGGCCAGGAC
CCAACGCTGCCCGAGATGCGCCGCGTGCGGCTGCTGGAGATGGCGGACGCGATGGATATGTTCTGCCAAGGGTTGGTTTGCG
CATTCACAGTTCTCCGCAAGAATTGATTGGCTCCAATTCTTGGAGTGGTGAATCCGTTAGCGAGGTGCCGCCGGCTTCCATTCA
GGTCGAGGTGGCCCGGCTCCATGCACCGCGACGCAACGCGGGGAGGCAGACAAGGTATAGGGCGGCGCCTACAATCCATGC
CAACCCGTTCCATGTGCTCGCCGAGGCGGCATAAATCGCCGTGACGATCAGCGGTCCAATGATCGAAGTTAGGCTGGTAAGAG
CCGCGAGCGATCCTTGAAGCTGTCCCTGATGGTCGTCATCTACCTGCCTGGACAGCATGGCCTGCAACGCGGGCATCCCGATG
CCGCCGGAAGCGAGAAGAATCATAATGGGGAAGGCCATCCAGCCTCGCGTCGCGAACGCCAGCAAGACGTAGCCCAGCGCG
TCGGCCGCCATGCCGGCGATAATGGCCTGCTTCTCGCCGAAACGTTTGGTGGCGGGACCAGTGACGAAGGCTTGAGCGAGG
GCGTGCAAGATTCCGAATACCGCAAGCGACAGGCCGATCATCGTCGCGCTCCAGCGAAAGCGGTCCTCGCCGAAAATGACCC
AGAGCGCTGCCGGCACCTGTCCTACGAGTTGCATGATAAAGAAGACAGTCATAAGTGCGGCGACGATAGTCATGCCCCGCGC
CCACCGGAAGGAGCTGACTGGGTTGAAGGCTCTCAAGGGCATCGGTCGAGATCCCGGTGCCTAATGAGTGAGCTAACTTACA
TTAATTGCGTTGCGCTCACTGCCCGCTTTCCAGTCGGGAAACCTGTCGTGCCAGCTGCATTAATGAATCGGCCAACGCGCGGG
GAGAGGCGGTTTGCGTATTGGGCGCCAGGGTGGTTTTTCTTTTCACCAGTGAGACGGGCAACAGCTGATTGCCCTTCACCGC
CTGGCCCTGAGAGAGTTGCAGCAAGCGGTCCACGCTGGTTTGCCCCAGCAGGCGAAAATCCTGTTTGATGGTGGTTAACGGC
GGGATATAACATGAGCTGTCTTCGGTATCGTCGTATCCCACTACCGAGATATCCGCACCAACGCGCAGCCCGGACTCGGTAATG
GCGCGCATTGCGCCCAGCGCCATCTGATCGTTGGCAACCAGCATCGCAGTGGGAACGATGCCCTCATTCAGCATTTGCATGGT
TTGTTGAAAACCGGACATGGCACTCCAGTCGCCTTCCCGTTCCGCTATCGGCTGAATTTGATTGCGAGTGAGATATTTATGCCA
GCCAGCCAGACGCAGACGCGCCGAGACAGAACTTAATGGGCCCGCTAACAGCGCGATTTGCTGGTGACCCAATGCGACCAG
ATGCTCCACGCCCAGTCGCGTACCGTCTTCATGGGAGAAAATAATACTGTTGATGGGTGTCTGGTCAGAGACATCAAGAAATAA
CGCCGGAACATTAGTGCAGGCAGCTTCCACAGCAATGGCATCCTGGTCATCCAGCGGATAGTTAATGATCAGCCCACTGACGC
GTTGCGCGAGAAGATTGTGCACCGCCGCTTTACAGGCTTCGACGCCGCTTCGTTCTACCATCGACACCACCACGCTGGCACCC
AGTTGATCGGCGCGAGATTTAATCGCCGCGACAATTTGCGACGGCGCGTGCAGGGCCAGACTGGAGGTGGCAACGCCAATC
AGCAACGACTGTTTGCCCGCCAGTTGTTGTGCCACGCGGTTGGGAATGTAATTCAGCTCCGCCATCGCCGCTTCCACTTTTTCC

CGCGTTTTCGCAGAAACGTGGCTGGCCTGGTTCACCACGCGGGAAACGGTCTGATAAGAGACACCGGCATACTCTGCGACAT
CGTATAACGTTACTGGTTTCACATTCACCACCCTGAATTGACTCTCTTCCGGGCGCTATCATGCCATACCGCGAAAGGTTTTGCG
CCATTCGATGGTGTCCGGGATCTCGACGCTCTCCCTTATGCGACTCCTGCATTAGGAAGCAGCCCAGTAGTAGGTTGAGGCCGT
TGAGCACCGCCGCCGCAAGGAATGGTGCATGCAAGGAGATGGCGCCCAACAGTCCCCCGGCCACGGGGCCTGCCACCATAC
CCACGCCGAAACAAGCGCTCATGAGCCCGAAGTGGCGAGCCCGATCTTCCCCATCGGTGATGTCGGCGATATAGGCGCCAGC
AACCGCACCTGTGGCGCCGGTGATGCCGGCCACGATGCGTCCGGCGTAGAGGATCGAGATCTCGATCCCGCGAAAT

Fig. S8. Plasmid pJKL1, pET -{PPAT, term, D2+D7}, 6974 bp. Vector Builder ID: VB221111-1136vyp. Plasmid map (above) and sequence (below) 5’ → 3’.

Table S1. RNA and oligo sequences

**Name**	**Sequence (5'→3')**
D2 RNA	AGGUGCGAAAGCACACAGAGUA
D3 RNA	AGGGUGCGAAAGCACACAGAGU
D4 RNA	AGGAGUGCGAAAGCACACAGAG
D5 RNA	AGGACGUGCGAAAGCACACAGA
D7 RNA	AGGACCAGUGCGAAAGCACACA
D10 RNA	AGGACCAAGCGUGCGAAAGCAC
Φ2.5 with +1A top	GCGTAATACGACTCACTATTAGG
D2 bottom	TACTCTGTGTGCTTTCGCACCTAATAGTGAGTCGTATTACGC
D3 bottom	ACTCTGTGTGCTTTCGCACCCTAATAGTGAGTCGTATTACGC
D4 bottom	CTCTGTGTGCTTTCGCACTCCTAATAGTGAGTCGTATTACGC
D5 bottom	TCTGTGTGCTTTCGCACGTCCTAATAGTGAGTCGTATTACGC
D7 bottom	TGTGTGCTTTCGCACTGGTCCTAATAGTGAGTCGTATTACGC
D10 bottom	GTGCTTTCGCACGCTTGGTCCTAATAGTGAGTCGTATTACGC
D2* RNA	**AG**GAGCAGACGAUUCGUCACACGUCCUGCUCUCGGCUGGAAAC***UAC UCA***UCAUCAUCGAGCUGGCAGUCGCAAAAAACCCCGCUUCGGCGGG GUUUUUUCGC
D7* RNA	**AGGACCA**GAGCAGACGAUUCGUCACACGUCCUGCUCUCGGCUGGAA AC***ACGUAA***UCAUCAUCGAGCUGGCAGUCGCAAAAAACCCCGCUUCGG CGGGGUUUUUUCGC
Forward Primer for HTS	AATGATACGGCGACCACCGAGATCTACACTCTTTCCCTACACGACGCTC TTCCGATCTGACGATTCGTCACACGTCCTGCTCTCGG
Reverse Primer 1 for HTS	CAGACGTGTGCTCTTCCGATCCCGAAGCGGGGTTTTTTGCGACTGCCA
Reverse Primer 2 for HTS	CAAGCAGAAGACGGCATACGAGATxxxxxxGTGACTGGAGTTCAGAC GTGTGCTCTTCC

Notes

1. The top strand oligo "Φ2.5 with +1A" was mixed with bottom strand oligos "D2 bottom – D10 bottom" to generate templates for D2-D10 RNA transcription. The T7 φ2.5 promoter sequence is marked by double underline and the initiating ATP is shown as **bolded green A**.

2. For 'D2* RNA' and 'D7* RNA' sequences, the ***bolded and italicized blue*** regions indicate the 6 nucleotide indices that differentiate these two transcripts apart from their different 5' end sequences (**bold)**. Yellow-highlighted segments were used as search queries of RNASeq data to identify whether a given amplicon originated from D2* or D7* RNA. Underlined regions of D2* and D7* RNAs indicate overlap with sequencing primers 'Forward Primer for HTS' and 'Reverse Primer 1 for HTS.' The latter of these overlaps with 'Reverse Primer 2 for HTS' (light blue) and appends the Illumina adapters and their respective sequencing indices. We used NEBNext Index (1-16) Primers for Illumina. 16 different 'Reverse Primer 2 for HTS' oligos (corresponding to the 16 indices; red 'x') were use in the second PCR for high-throughput sequencing preparation. Index sequences are from the instruction manual for the NEBNext Multiplex Small RNA Library Prep Set 1, Set 2, Index Primers 1-48 and Multiplex Compatible (https://rb.gy/0dbqe9).

Table S2. High-throughput sequencing raw data and processing for total isolated RNA samples

**Total Isolated RNA Samples**	**+I 1.1**	**+I 1.2**	**+I 2.1**	**+I 2.2**	**-I 3.1**	**-1 3.2**	**-I 4.1**	**-I 4.2**
Raw total reads	142	27,205	19,775	30,591	265	1,281	11,952	926
Discarded Long reads (>29nt)	130	3,075	1,600	3,893	259	1,218	1,879	601
Discarded Short reads (<23nt)	-	14	6	14	-	2	2	3
Total processed sequence reads	12	24,116	18,169	26,684	6	61	10,071	322
D2* + D7* RNA Total Counts	11	23,210	17,513	25,738	6	58	9,668	307
D2* RNA	9	18,674	13,610	20,088	4	42	7,626	246
D7* RNA	2	4,536	3,903	5,650	2	16	2,042	61
Percent of D2* RNA	81.8	80.5	77.7	78.1	66.7	72.4	78.9	80.1
Percent of D7* RNA	18.2	19.5	22.3	22.0	33.3	27.6	21.1	19.9

Note. Total isolated RNA samples were prepared without sulfur fractionation. Data processing was performed using cutadapt to trim the 5' and 3' constant regions from sequences and to discard any uncut sequences or sequences with lengths not within ± 3 nt of the expected size (26 nt) after trimming. 'Raw total reads' is the number of sequences prior to any processing, 'Discarded' long and short sequences did not fit within the ± 3 nt parameter, and the 'Total processed sequence reads' were analyzed using FASTAptameR2.0. Of the total processed reads, those that contained the index sequence for D2* or D7* RNA were identified and counted. 'Percent of D2* RNA' and 'Percent of D7* RNA' were determined by dividing the individual D2* or D7* counts by the total number of (D2* + D7*) counts. Column headers indicate sources of each RNA sample. '+I' indicates plus induction and '-I' indicates no induction. The first number indicates which culture (1-4) the sample was isolated from, and the second number indicates which technical replicate the sample came from. For instance, sample "+I 1.2" was from the second technical replicate of the first culture that was induced to express PPAT. Columns shown in light gray indicate samples with fewer than 2,000 total unique processed reads and are not plotted in fig 3 of main manuscript.

Table S3. High-throughput sequencing raw data and processing for sulfur partitioned RNA samples

**Sulfur Partitioned Samples**	**+I 1.1**	**+1 1.2**	**+I 2.1**	**+1 2.2**	**-I 3.1**	**-1 3.2**	**-I 4.1**	**-I 4.2**
Raw total reads	19,444	25,809	27,779	945	8,658	17,253	806	18,960
Discarded Long reads (>29nt)	3,050	3,163	3,556	933	1,343	6,550	804	3,058
Discarded Short reads (<23nt)	8,618	8,288	3,927	0	6,177	8,262	0	4,243
Total processed sequence reads	7,776	14,358	20,296	12	1,138	2,441	2	11,659
D2* + D7* RNA Total Counts	6,081	12,112	18,502	11	322	1,564	2	10,873
D2* RNA	1,136	2,303	7,415	11	183	599	1	2,856
D7* RNA	4,945	9,809	11,087	0	139	965	1	8,017
Percent of D2* RNA	18.7	19.0	40.1	100.0	56.8	38.3	50.0	26.3
Percent of D7* RNA	81.3	81.9	60.0	0.0	43.2	61.7	50.0	73.7

Note. Data were processed and analyzed as in **Table S2**.

References

1. Sapkota K & Huang F (2018) Efficient one-pot enzymatic synthesis of dephospho coenzyme A. *Bioorg Chem* 76:23-27.
2. Strauss E & Begley TP (2002) The antibiotic activity of N-pentylpantothenamide results from its conversion to ethyldethia-coenzyme a, a coenzyme a antimetabolite. *J Biol Chem* 277(50):48205-48209.
3. Brand LA & Strauss E (2005) Characterization of a new pantothenate kinase isoform from Helicobacter pylori. *J Biol Chem* 280(21):20185-20188.
4. Miller JR*, et al.* (2007) Phosphopantetheine adenylyltransferase from Escherichia coli: investigation of the kinetic mechanism and role in regulation of coenzyme A biosynthesis. *J Bacteriol* 189(22):8196-8205.
5. Sousa R & Padilla R (1995) A mutant T7 RNA polymerase as a DNA polymerase. *EMBO J* 14(18):4609-4621.
6. Zuker M (2003) Mfold web server for nucleic acid folding and hybridization prediction. *Nucleic Acids Res* 31(13):3406-3415.
7. Ivanetich KM & Goold RD (1989) A rapid equilibrium random sequential bi-bi mechanism for human placental glutathione S-transferase. *Biochim Biophys Acta* 998(1):7-13.
8. Lee HJ*, et al.* (2011) Structural and kinetic analysis of substrate binding to the sialyltransferase Cst-II from Campylobacter jejuni. *J Biol Chem* 286(41):35922-35932.
9. Segel IH (1975) Enzyme Kinetics: Behaviour and Analysis of Rapid Equilibrium and Steady-State Enzyme Systems. *John Wiley & Sons, Inc., New York.*
10. Cleland WW (1963) The kinetics of enzyme-catalyzed reactions with two or more substrates or products. I. Nomenclature and rate equations. *Biochim Biophys Acta* 67:104-137.
11. McEntee K, Weinstock GM, & Lehman IR (1980) recA protein-catalyzed strand assimilation: stimulation by Escherichia coli single-stranded DNA-binding protein. *Proc Natl Acad Sci U S A* 77(2):857-861.
12. Biondi E & Burke DH (2012) Separating and analyzing sulfur-containing RNAs with organomercury gels. *Methods Mol Biol* 883:111-120.
13. Ditzler MA*, et al.* (2013) High-throughput sequence analysis reveals structural diversity and improved potency among RNA inhibitors of HIV reverse transcriptase. *Nucleic Acids Res* 41(3):1873-1884.
14. Martin M (2011) Cutadapt removes adapter sequences from high-throughput sequencing reads. *EMBnet. journal* 17:10–12.
15. Alam KK, Chang JL, & Burke DH (2015) FASTAptamer: A Bioinformatic Toolkit for High-throughput Sequence Analysis of Combinatorial Selections. *Mol Ther Nucleic Acids* 4(3):e230.
16. Kramer ST, Gruenke PR, Alam KK, Xu D, & Burke DH (2022) FASTAptameR 2.0: A web tool for combinatorial sequence selections. *Mol Ther Nucleic Acids* 29:862-870.
